# Unlocking the potential of alkaline polymethacrylates via ionic crosslinking in amorphous solid dispersions

**DOI:** 10.1016/j.ijpx.2026.100531

**Published:** 2026-04-01

**Authors:** Lukas Mild, Alicia Stakemeier, My Ngoc Nguyen Thi, Karl G. Wagner

**Affiliations:** a Department of Pharmaceutical Technology and Biopharmaceutics, University of Bonn, Gerhard-Domagk-Strasse 3, 53121 Bonn, Germany; b Kekulé-Institut für Organische Chemie und Biochemie, University of Bonn, Gerhard-Domagk-Strasse 1, 53121 Bonn, Germany

**Keywords:** Amorphous solid dispersion (ASD), Hot-melt extrusion (HME)., Drug-polymer miscibility., Supersaturation maintenance., Vacuum compressing molding (VCM)., Polymethacrylates (Eudragit E)., Poorly water-soluble drugs (BCS Class II).

## Abstract

Amorphous solid dispersions (ASDs) are a well-established strategy to enhance the oral bioavailability of poorly water-soluble drugs; however, their broader application remains limited by drug–polymer compatibility and pronounced pH-dependent performance. In this study, alkaline polymethacrylates (Eudragit® E) are investigated as versatile carrier systems for ASDs targeting poorly water-soluble BCS Class II drugs, using acid-modified formulations to enable functionality under physiological conditions. ASDs were prepared by hot-melt extrusion (HME) and vacuum compression molding (VCM) to assess process robustness, drug–polymer miscibility, thermal behavior, and dissolution performance. Solid-state characterization confirmed complete amorphization and stable glass formation across multiple drug loadings. The presence of organic acids induced controlled protonation of the polymer, giving rise to an ionically crosslinked polymer–acid network with distinct thermal signatures and enhanced structural stability. Dissolution studies demonstrated rapid generation and prolonged maintenance of supersaturation in phosphate buffer at pH 6.8, significantly exceeding the thermodynamic solubility of the investigated drugs. Supersaturation behavior correlated strongly with the calculated degree of polymer protonation rather than with acid content alone, indicating that protonation-controlled polymer solubilization governs dissolution performance. Long-term stability testing further demonstrated preserved amorphous structure and consistent dissolution behavior under both standard and accelerated storage conditions. Overall, this work establishes acid-modified alkaline polymethacrylates as a broadly applicable platform for ASD formulation. By decoupling solubility enhancement from specific drug chemistries through polymer-driven ionic crosslinking, this approach expands the design space of ASDs and enables the rational development of stable, high-performance formulations for poorly water-soluble drugs.

## Highlights

• Alkaline polymethacrylates enabled stable ASDs for poorly soluble BCS II drugs.
• Acid–base structured Eudragit® E ASDs sustain supersaturation at physiological pH.
• Drug–polymer miscibility governed thermal stability and dissolution behavior.
• HME and VCM enable robust processing of ionically structured ASD formulations.
• A platform approach expands ASD design beyond drug-specific interactions.

## Graphical abstract

**Figure:**
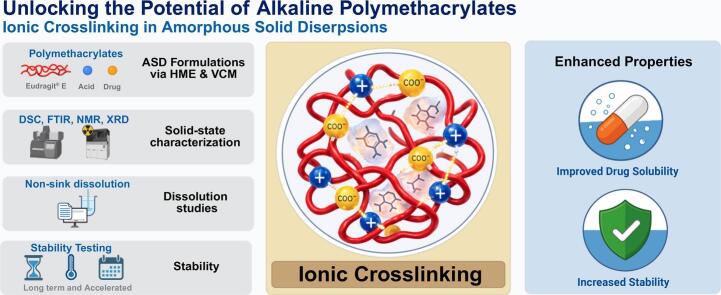

## Abbreviations / Glossary

**Table:** 

AA	adipic acid
API	active pharmaceutical ingredient
ASD	amorphous solid dispersion
CA	citric acid
CXB	celecoxib
COSY	correlated spectroscopy
DL	drug load
DMAE	dimethylaminoethyl
DMSO	dimethyl sulfoxide
EFZ	efavirenz
EPO	Eudragit® E
FaSSIF-V2	Fasted State Simulated Intestinal Fluid V2
HME	hot-melt-extrusion
KTZ	ketoconazole
TA	tartaric acid
VCM	vacuum compressing molding

## 1 Introduction

Poor aqueous solubility remains a key obstacle in oral drug development, roughly 40% of marketed drugs and up to 90% of new chemical entities show poor aqueous solubility (Nyamba et al., 2024). Amorphous solid dispersions (ASDs) address this limitation by enhancing dissolution performance of poorly soluble drugs (Mullins and Macek, 1960; Sekiguchi and Obi, 1961; Chiou and Riegelman, 1971; Doherty and York, 1987; Forster et al., 2001). In ASDs, the drug is molecularly dispersed in an amorphous, high-energy polymer matrix (El-Hinnawi and Najib, 1987; Taylor and Zografi, 1997). This configuration increases apparent solubility compared to the crystalline form (Miller et al., 2012; Dahan et al., 2013; Alonzo et al., 2011; Sun and Lee, 2015; Ueda et al., 2025). In many ASD systems, the polymer fulfills a dual role: it promotes amorphization in the solid state and stabilizes supersaturation during dissolution, often described as the “spring and parachute” model (Brewster et al., 2008). However; often a polymer facilitating ASD formation in solid state is ineffective at stabilizing supersaturation in liquid state. In such cases ternary ASDs including two polymers have been successfully introduced (Augustijns and Brewster, 2012; Hirlak et al., 2021; Monschke and Wagner, 2020; Pöstges et al., 2022).

Among single polymer ASDs, Eudragit E® (EPO), a cationic copolymer of dimethylaminoethyl(DMAE)methacrylate, butyl-methacrylate and methyl-methacrylate (2:1:1), has gained considerable interest as a carrier in ASDs (Frank et al., 2022; Jeganathan and Prakya, 2015; Lin et al., 2018; Xie et al., 2017). Originally developed for taste masking, EPO is soluble only at pH < 5 due to protonation of its tertiary amines (dos Santos et al., 2021). Consequently; EPO readily dissolves in the acid gastric environment but becomes insoluble upon transition to the near-neutral pH of the small intestine. This pronounced pH dependency distinguishes EPO from more commonly applied ASD polymers such as HPMCAS or PVPVA; which are designed to dissolve under intestinal conditions and therefore dominate clinically established ASD formulations (Moseson et al., 2024). Incorporation of acidic drugs allows EPO to remain accessible under physiological conditions; enabling partial ionization that promotes strong ionic interactions and dissolution of the ASD in the entire physiological pH range. Moreover; these interactions enhance solubility and prolong supersaturation and; in some systems; reduce precipitation (Neusaenger et al., 2023). However; this strategy remains limited to strongly acidic drugs; and alternative approaches are required for neutral or weakly basic compounds (Fine-Shamir and Dahan, 2019; Saal et al., 2017; Xie et al., 2017). However, the strong ionic potential of EPO also makes it prone to interactions with physiological components, such as bile colloids, altering their structure and potentially reducing the intestinal flux of bile-associated drugs (Schlauersbach et al., 2022b; Schlauersbach et al., 2021), which may decrease systemic exposure in vivo (Schlauersbach et al., 2022a). Such effects are not restricted to bile-associated drugs; strong interactions between alkaline polymethacrylates and basic drugs (including cation–π interactions) have also been reported to shift release toward sustained profiles and slightly compromise exposure in vivo (Saal et al., 2018). Moreover; excipient interactions can reduce oral bioavailability in other challenging modalities; as shown for highly lipophilic PROTACs (Hofmann et al., 2025). Separately; medium properties such as buffer capacity can significantly influence dissolution kinetics and supersaturation profiles; independent of drug “complexity” (Bapat et al., 2023). Taken together; EPO offers attractive features as an ASD carrier (strong ionic interaction potential and efficient amorphization); but its pronounced pH-dependent solubility limits consistent intestinal performance. Consequently; alkaline polymethacrylates such as EPO are rarely used in clinically relevant ASD formulations compared with commonly employed carriers such as Hydroxypropyl methylcellulose acetate succinate (HPMCAS) or Polyvinylpyrrolidone(PVP)-based polymers (Moseson et al., 2024). These limitations highlight the need for formulation strategies that enable controlled polymer protonation independently of drug acidity, thereby broadening the applicability of EPO-based ASDs.

One promising approach employs acidic pH modifiers that generate local micro acidification within the ASD. Such additives can partially protonate EPO's DMAE groups, maintaining polymer solubility even near neutral pH, as shown for the combination of phenytoin/EPO with Saccharin (Ueda et al., 2018a). Related concepts of generating polyelectrolyte-like matrices prior to thermal processing have also been described; for example by combining a solvent-based pre-step with subsequent HME to enable ionic polymer functionality (Ditzinger et al., 2019).This localized protonation mechanism offers a feasible strategy to achieve pH-independent release while preserving precipitation inhibition. Similar strategies involving the incorporation of ionizable *co*-formers have been successfully investigated in combination with various other ASD polymers. For instance; in HPMCAS-based systems; the addition of organic acids has been shown to modulate the polymer's ionization state and dissolution rate; while in Copovidone matrices; the use of salt forms or ionic surfactants was demonstrated to significantly enhance physical stability and modify supersaturation profiles (Duong et al., 2022). Supersaturation maintenance is closely linked to the ability of polymers to engage in drug–polymer interactions that suppress nucleation and crystal growth (Baird and Taylor, 2012; Ilevbare et al., 2013; Indulkar et al., 2019; Que et al., 2021). Beyond bulk solubility, ionization-controlled interfacial phenomena, including polymer solubilization and chain disentanglement, have emerged as key determinants of dissolution behavior and crystallization outcomes (Bapat et al., 2024; Hiew et al., 2022). These studies highlight that supersaturation maintenance is dictated not only by the rate of polymer dissolution but also by molecular-level interactions and their specific influence on crystallization kinetics.

Yet, systematic studies on protonated EPO-based ASDs remain limited, and key mechanistic aspects, particularly the interplay between microenvironmental pH, polymer protonation and supersaturation stability are not fully understood. In particular, it is essential to disentangle whether enhanced performance arises simply from acid-enhanced polymer solubilization (enabling matrix dissolution) or from genuine stabilization of supersaturated drug states under intestinal conditions (Bapat et al., 2024). Additionally; the implications of protonation-driven systems for pharmaceutical processability and translation; across solvent-based routes and melt-based manufacturing; remain insufficiently clarified. This work therefore investigates the influence of the acidic pH modifiers; adipic acid (AA); citric acid (CA); and tartaric acid (TA); on the performance of EPO-based ASDs. To systematically evaluate the robustness of the acid-modified EPO platform; three model drugs representing different challenges in ionizable ASD design were selected. Celecoxib (CXB; pK_a_ = 9.5; weak acid) (Lubach et al., 2013) and Efavirenz (EFZ; pK_a_ = 10.2; weak acid; largely unionized at physiological pH) (Adeola et al., 2021) were chosen to investigate whether microenvironmental acidification can compensate for the lack of intrinsic drug–polymer ionic interactions. Under intestinal conditions; both compounds remain predominantly non-ionized and therefore cannot promote polymer protonation. In contrast; Ketoconazole (KTZ; pK_a_ = 6.7; weak base) (Neuwirth et al., 2023) was selected to evaluate the platform under conditions of potential electrostatic competition for protons between the cationic drug and the cationic polymer. This scenario is of particular interest as recent mechanistic studies emphasize how electrostatic repulsion and charge-induced polymer disentanglement can significantly dictate drug release and matrix erosion (Bapat et al., 2024; Hiew et al., 2022).

By systematically elucidating underlying molecular interactions and linking them to the pH-dependent polymer behavior during dissolution, we provide insight into how protonation, microenvironmental acidification, and drug–polymer interactions govern the generation and maintenance of supersaturated states under physiologically relevant conditions. Integrating solid-state and dissolution-based characterization, this study establishes a mechanistic framework for the rational design of functionally pH-independent EPO-based ASDs, and thereby contributing to unlocking the full potential of alkaline polymers for oral drug delivery.

## 2 Material and methods

### 2.1 Materials

Eudragit® E PO (EPO) was kindly provided by Evonik (Darmstadt, Germany). CXB and KTZ were obtained from Swapnroop Drugs & Pharmaceuticals (Aurangabad, India), and Efavirenz from Tokyo Chemical Industry (Tokyo, Japan). AA was purchased from Fisher Scientific (Geel, Belgium), anhydrous CA from VWR Chemicals (Leuven, Belgium), and TA was kindly provided from Boehringer Ingelheim (Ingelheim am Rhein, Germany). The FaSSIF-V2-medium was prepared according to (Denninger et al., 2020). All solvents are of analytical grade or higher.

### 2.2 Preparation of ASD Formulations using Vacuum Compressing Molding (VCM) and Hot-Melt-Extrusion (HME)

#### 2.2.1 Screening for the ideal citric acid concentration

To determine the optimal acid concentration, VCM samples were produced. EPO, AA/CA/TA, and CXB were homogenized in a ball mill (MM400, Retsch GmbH, Haan, Germany) at 30 Hz for 5 min three times each. The repeated milling cycles were applied to ensure homogeneous pre-distribution of the organic acids within the polymer matrix while minimizing excessive mechanical stress. Subsequently, 250–500 mg of the premixed powder was transferred to a VCM tool (MeltPrep GmbH, Graz, Austria) and processed under vacuum at 163 °C (AA & CA); 172 °C (TA). The VCM process was characterized by a rapid heating phase, ensuring that the target temperature was maintained for only a very short duration, comparable to the residence time in a hot-melt extruder. Samples were processed for two consecutive 5 min heating cycles to ensure complete melting of CXB and the formation of an ASD. Between the cycles, the VCM tool was transferred to a cooling station and allowed to cool completely to room temperature. This iterative melting–cooling approach minimized cumulative thermal stress on the organic acids while promoting phase homogenization. The solidified samples were briefly reground (MM400, Retsch GmbH; <30 s, 30 Hz) and subsequently passed through a 355 μm sieve. The passage of the VCM produced samples, were used solely for the systematic determination of the ideal pH-modifier concentration. An overview of all formulations is provided in Table 1.

**Table 1:** Comprehensive formulation table of the binary EPO–API and ternary EPO–acid–API ASDs investigated in this study.

Formulation code	API	Acid	DL (% w/w)	Acid in (EPO + acid) (%)^a^	EPO (% w/w)	Acid (% w/w)	API (% w/w)
**Binary EPO–API ASDs**							
EPO-API	CXB / EFZ / KTZ	–	10	0	90.0	0	10
	CXB / EFZ / KTZ	–	20	0	80.0	0	20

**Ternary EPO–acid–API ASDs (fixed acid fraction)**							
EPO-CA-API	CXB / EFZ / KTZ	CA	10	10	81.0	9.0	10
	CXB / EFZ / KTZ	CA	20	10	72.0	8.0	20
EPO-AA-API	CXB / EFZ / KTZ	AA	10	10	81.0	9.0	10
	CXB / EFZ / KTZ	AA	20	10	72.0	8.0	20
EPO-TA-API	CXB / EFZ / KTZ	TA	10	10	81.0	9.0	10
	CXB / EFZ / KTZ	TA	20	10	72.0	8.0	20

**Neutralization / acid-content series (CXB)**							
EPO-Acid-CXB	CXB	CA / AA / TA	10	5	85.5	4.5	10
	CXB	CA / AA / TA	10	10	81.0	9.0	10
	CXB	CA / AA / TA	10	15	76.5	13.5	10
	CXB	CA / AA / TA	10	20	72.0	18.0	10

a Acid contents were calculated relative to the polymer–acid fraction (EPO + acid). Thus, the DL was subtracted from the total formulation mass (100%), and the specified acid percentage was applied to the remaining fraction.

#### 2.2.2 Two-stage production of the final ASD formulations

A two-stage manufacturing process was used for the final formulations. First, EPO and CA were processed in a ratio of 80:20% (*w*/w) using hot melt extrusion (ThreeTec ZE12, ThreeTec GmbH, Seon, Switzerland) at max. 145 °C to form a homogeneous polymer blend. This procedure is based on the approach described by (Pöstges et al., 2023; Pöstges et al., 2022) to ensure phase homogeneity of the organic acid in the polymethacrylate carrier. The extrudate strand was subsequently ground, sieved and stored in a desiccator. In a second step, the polymer-blend was homogenized with the API at a drug loading (DL) of 10% or 20% (w/w) in a ball mill (30 Hz, 3 × 5 min). The powders were further processed using the VCM-tool under vacuum at 140 °C (EFZ), 150 °C (KTZ) and 163 °C (CXB) for 3–4 heating cycles of 5 min each, using the same melting–cooling procedure described above to promote phase homogenization while limiting thermal exposure. The high piston pressure applied by the VCM tool additionally suppressed volatilization of the organic acids at elevated temperatures, thereby ensuring the maintenance of the intended stoichiometric acid-to-polymer ratio, which was verified via HPLC. After milling (MM400, <30 s, 30 Hz), the samples were passed through a 355 μm sieve and the passage stored in a desiccator until further analysis to prevent moisture absorption. The organic acid content was calculated relative to the polymer–acid fraction (EPO + Acid). Consequently, the drug loading was subtracted from the total formulation mass (100%), and the desired acid percentage was applied to the remaining fraction.

### 2.3 X-Ray Powder Diffraction (XRPD)

Neat API, pH modifiers, and milled ASDs were analyzed in reflection mode over a 2θ range of 4–50° with a step size of 0.017° 2θ using an X'Pert MRD Pro diffractometer (PANalytical, Almelo, Netherlands). Measurements were conducted with an X'Celerator detector and nickel-filtered Cu Kα1 radiation at 45 kV and 40 mA. To ensure sufficient sensitivity for the detection of trace crystallinity (estimated detection limit 1%), a step size of 0.017° 2θ and a scan speed of 0.042°/s were applied, resulting in a total acquisition time of 19 min per sample. For CXB, all three acidic coformers were examined to evaluate the generalizability of the EPO–acid platform. For EFZ and KTZ, only EPO-CA ASDs were analyzed to assess amorphization status. Precipitates obtained from the precipitation assay were additionally analyzed in transmission geometry over a 2θ range of 5–42° using the same instrumental setup. Bragg peak assignments were based on comparison to experimentally measured crystalline API reference diffractograms acquired under identical instrumental conditions. Reference patterns were additionally cross-checked against published diffraction data and reported crystal structures (Cuffini et al., 2009; Dhadphale and Donde, 2025; Momma and Izumi, 2011; Peeters et al., 2004). All measurements were performed in triplicate (*n* = 3).

### 2.4 Differential Scanning Calorimetry (DSC)

Thermal analysis was performed with the neat APIs, pre-extrudate EPO-CA and the final ASDs by employing a DSC 2 instrument (Mettler-Toledo, Gießen, Germany) equipped with a nitrogen cooling system. Approximately 10 mg of each sample was precisely weighed into aluminum pans, which were sealed with pierced lids. Measurements were conducted in TOPEM mode, a multi-frequency temperature-modulated program, applying a heating rate of 2 K/min from 0 °C to 180 °C for AA and to 120 °C for CA/TA with a temperature pulse amplitude of ±0.5 °C under a constant nitrogen purge (30 mL/min). All measurements were performed in triplicate (n = 3) and are reported as mean ± standard deviation. For binary and ternary mixtures, Tg values were additionally evaluated using the Gordon–Taylor equation to assess miscibility and deviations from ideal mixing behavior.

### 2.5 Fourier-Transform Infrared Spectroscopy (FT-IR)

To investigate potential molecular interactions in the solid state, FT-IR spectroscopy was performed using a Spectrum Two instrument (PerkinElmer, Waltham, MA, USA). Spectra were recorded in the range of 450–4000 cm^−1^ for raw materials, physical mixtures, polymer-based placebo formulations (EPO-CA), and amorphous solid dispersions (EPO-CA-API).

### 2.6 ^1^H-Nuclear Magnetic Resonance Spectroscopy (^1^H-NMR)

1H-NMR was recorded on the Bruker Avance I 400 MHz, Bruker Avance III HD Ascend 500 MHz Prodigy and Bruker Avance III HD Ascend 700 MHz Cryo. Chemical shifts are given in parts per million (ppm) and referenced to the residual ^1^H signals in deuterated solvents. All NMR spectra were recorded at room temperature. The NMR samples were prepared and recorded under ambient conditions. DMSO‑*d*_6_ and acetone‑*d*_6_ (Deutero GmbH, Kastellaun, Germany) were used for the preparation of the NMR samples. NMR data analysis was performed using MestReNova 14.2.1 (Mestrelab Research, Santiago de Compostela, Spain).

### 2.7 Monophasic Non-Sink Dissolution

Non-sink dissolution testing was performed over 180 min using a miniaturized USP dissolution apparatus II (MiniDissolution apparatus) (Zecevic and Wagner, 2013). The temperature was maintained at 37 °C and the paddle speed set to 75 rpm. A total of 40 mg of either physical mixtures or ASDs formulated with DL of 10% or 20% (*w*/w) and varying ratios of pH modifiers was dissolved, resulting in an API concentration of 200 μg/mL in 0.05 M phosphate buffer (PBS) or FaSSIF-V2 (adjusted to pH 6.8). All dissolution experiments were conducted in triplicate (*n* = 3) and are reported as mean ± SD. API concentrations were monitored online using an 8453 UV/VIS spectrophotometer (Agilent, Waldbronn, Germany), with real-time correction for light scattering.

### 2.8 Two-stage dissolution

Two-stage pH-shift dissolution experiments were performed for CXB formulations to simulate gastric-to-intestinal transition. In stage 1, samples were dispersed in 0.1 M HCl (pH 1.2) at 37 °C for 30 min under identical hydrodynamic conditions (75 rpm). Subsequently, the medium was adjusted to pH 6.8 by addition of concentrated phosphate buffer according to (Denninger et al., 2020)**,** yielding final intestinal simulation conditions identical to monophasic experiments. Concentrations were monitored for a total of 180 min. All experiments were conducted in triplicate (n = 3) and are reported as mean ± SD.

### 2.9 High Performance Liquid Chromatograpgy (HPLC) – solubility determination and acid retention

The thermodynamic solubility of the investigated APIs was determined by adding an excess amount of crystalline drug to 0.05 M sodium phosphate buffer (pH 6.8) and equilibrating the suspension at 37 °C for 48 h under continuous agitation. After equilibration, the supernatant was separated from undissolved solids by centrifugation at 15000 rpm for 15 min. The clear supernatant was carefully collected and filtered through a 0.22 μm membrane filter prior to analysis. Drug concentrations were quantified by HPLC as described below. For the determination of acid retention after thermal processing, the content of AA, CA, and TA in processed ASDs was quantified by HPLC to verify that the intended stoichiometric acid-to-polymer ratio was maintained. Samples were dispersed in 0.05 M phosphate buffer adjusted to pH 2.6 using phosphoric acid, centrifuged at 15000 rpm for 15 min, and the clear supernatant was carefully collected and filtered (0.22 μm) prior to analysis. Chromatographic separation was performed using an Agilent Series 1100 HPLC system (Agilent, Waldbronn, Germany) equipped with a Nucleosil C18 column (5 μm, 4.6 × 250 mm, Macherey-Nagel GmbH & Co. KG, Düren, Germany). For organic acid analysis, the mobile phase consisted of 0.05 M phosphate buffer (pH 2.6) applied at a flow rate of 1.0 mL/min, and detection was carried out at 210 nm. For API quantification, an adapted mobile phase consisting of acetonitrile/water (70/30, *v*/v) was used. For KTZ, the aqueous phase was adjusted to acidic pH to improve solubility and peak shape. Detection wavelengths were 253 nm for celecoxib, 248 nm for efavirenz, and 243 nm for ketoconazole. External calibration curves were established over concentration ranges of 1–100 μg/mL for organic acids, 0.1–20 μg/mL for CXB and 1–50 μg/mL for EFZ and KTZ. Linearity was confirmed with correlation coefficients R^2^ ≥ 0.999. Calibration standards for acid determination were prepared in the presence of EPO (20/80 *w*/w) to account for potential matrix effects. Acid retention was calculated as the ratio of experimentally determined to theoretical acid content and is reported as mean ± one standard deviation (*n* = 3).

### 2.10 Turbidity measurements

Turbidity was monitored in-line using the same UV/VIS spectrophotometer (8453, Agilent, Waldbronn, Germany) employed for dissolution analysis. Light scattering was measured at 830 nm, a wavelength at which none of the investigated APIs exhibit absorbance, thereby allowing selective detection of turbidity without interference from drug absorption. Measurements were performed under identical hydrodynamic conditions as the dissolution experiments (MiniDissolution apparatus, 37 °C, 75 rpm). Placebo formulations (EPO and EPO-CA without API) were analyzed separately to assess polymer-associated turbidity contributions. All measurements were conducted in triplicate (n = 3).

### 2.11 Particle size analysis during dissolution

Particle size distributions were determined using a Horiba LA-960 laser diffractometer (Horiba Ltd., Kyoto, Japan). Approximately 15 mL of 0.05 M phosphate-buffer was filled into a quartz cuvette. The samples were dispersed in the medium under continuous magnetic stirring to ensure a homogeneous suspension throughout the measurement. Particle size distributions were recorded from the same suspension at predefined time points (0, 5, 10, 15, 30, and 60 min) to monitor the time-dependent evolution of the particle population during dissolution. A red laser diode with a wavelength of 650 nm (5 mW) and a blue light-emitting diode with a wavelength of 405 nm (3 mW) were used as light sources. Particle size distributions were calculated using the Mie scattering model. All measurements were performed in triplicate (n = 3).

### 2.12 Precipitate analysis after supersaturation

To analyze potential precipitation of weakly soluble drugs under supersaturated conditions, a slurry-based supersaturation experiment was performed analogous to (Pöstges et al., 2022). A 40 mg/mL API stock solution was prepared in DMSO. Subsequently, 100 μL of this stock solution was injected into 20 mL dissolution medium (0.05 M phosphate buffer, pH 6.8, (or FaSSIF-V2), yielding an initial API concentration of 200 μg/mL corresponding to a fully molecularly dissolved state at *t* = 0. The experiments were conducted at 37 °C under identical hydrodynamic conditions as the dissolution studies (MiniDissolution apparatus, 75 rpm). After 180 min, the suspension was transferred into centrifuge tubes and centrifuged at 15,000 rpm for 15 min at room temperature. The supernatant was carefully removed, and the wet pellet was immediately subjected to transmission XRPD analysis without prior drying in order to minimize potential crystallization or polymorphic transformation during solvent removal.

### 2.13 Calculation of the maximum neutralization degree

For all ternary EPO–acid–API formulations, the neutralization degree was expressed as the ratio of acid-derived proton equivalents to tertiary amine sites of EPO. Moles of tertiary amine functionalities $n_{\text{DMAE}}$ were calculated from the EPO mass as $n_{\text{DMAE}}$ (mmol) = $m_{\mathrm{EPO}}$ (g) · 3.2 mmol/g, based on the supplier-reported alkali value (KOH value; Supplement, Table S1). Moles of acid ($n_{\text{acid}}$) were obtained from the respective acid mass and molar mass. The number of proton equivalents was then calculated as $z\cdot n_{\text{acid}}$, where z is the maximum number of dissociable protons (z=3 for citric acid, z=2 for adipic and tartaric acid). The neutralization degree α (1) was finally given by

(1) $$\alpha =\frac{z\cdot n_{\text{acid}}}{n_{\text{DMAE}}}$$

and is reported in Table S2 (Supplementary material) as proton equivalents per tertiary amine group (H^+^ equiv./NR_3_). The use ofz=3for citric acid represents a stoichiometric upper-bound approximation of its protonation capacity. Proton transfer from the organic acids to tertiary amine groups of EPO was rationalized based on acid–base equilibria. EPO exhibits a pK_a_ of approximately 7.3 (Obeidat et al., 2015) for its protonated dimethylaminoethyl groups. According to the Henderson–Hasselbalch relationship; at pH 5.5 the ratio [NR_3_]/[NR_3_H^+^] equal 10^(5.5–7.3)^ ≈ 0.016; corresponding to more than 98% protonation of the tertiary amine sites. This indicates that near-stoichiometric protonation (approximately one proton per NR_3_ group) is sufficient to render EPO highly ionized and soluble. As a qualitative descriptor of the acid–base driving force; the pK_a_ difference ΔpK_a_ between protonated EPO and the organic acids was considered; defined as ΔpK_a_ = pK_a_/(EPOH^+^) – pK_a_ (acid); using apparent pK_a_ (EPOH^+^) ≈ 7.3. Positive ΔpK_a_ values indicate thermodynamically favorable proton transfer from the acid to EPO. It should be noted that the dielectric environment in amorphous matrices differs markedly from aqueous media; such that pK_a_ values and ionization equilibria can be shifted and may be kinetically constrained. Accordingly; α is used here as a stoichiometric descriptor rather than as an absolute measure of ionization in an aqueous sense. In the present study; all acids exhibited sufficiently large ΔpK_a_ values to ensure efficient protonation of EPO; such that ΔpK_a_ primarily serves as a permissive criterion rather than a quantitative predictor of dissolution behavior. Citric acid is triprotic; with pK_a1_ = 3.13; pK_a2_ = 4.76; and pK_a3_ = 6.40 (Silva et al., 2009). While the first and second dissociation steps are strongly favored under these conditions, the third dissociation step is thermodynamically weaker and may be only partially accessed, particularly in a glassy polymer matrix with limited molecular mobility. Nevertheless, accounting for all three dissociable protons provides a conservative estimate of the maximum proton supply available for EPO protonation and enables consistent stoichiometric comparison across formulations. Although dissolution experiments were conducted in phosphate buffer at pH 6.8, a pH value of 5.5 was used here as a mechanistic reference for the microenvironmental pH at the dissolving polymer–water interface. This concept does not imply a change in bulk pH but reflects localized acidic conditions arising from organic acid release and polymer protonation at the particle surface, which govern EPO ionization and dissolution behavior.

### 2.14 Stability

Stability studies were conducted under temperature conditions in accordance with ICH guideline Q1A(R2). (ICH, 2003). Approximately 500 mg of EPO-based ASDs containing acidic coformers (AA, CA, TA) and 10% or 20% DL CXB were filled into type I glass vials with screw-cap closure equipped with 2 g silica gel desiccant and stored for six months at 25 °C / 60% relative humidity (RH). For accelerated stability testing, additional EPO-CA-CXB, -EFZ, and -KTZ were stored at 40 °C / 75% RH for six months (CXB) and one month (EFZ and KTZ). Physical stability was assessed by XRPD, while dissolution behavior was evaluated as described in Section 3.7.

## 3 Results

### 3.1 Solid-state of the ASDs

#### 3.1.1 XRPD

**Fig. 1:**
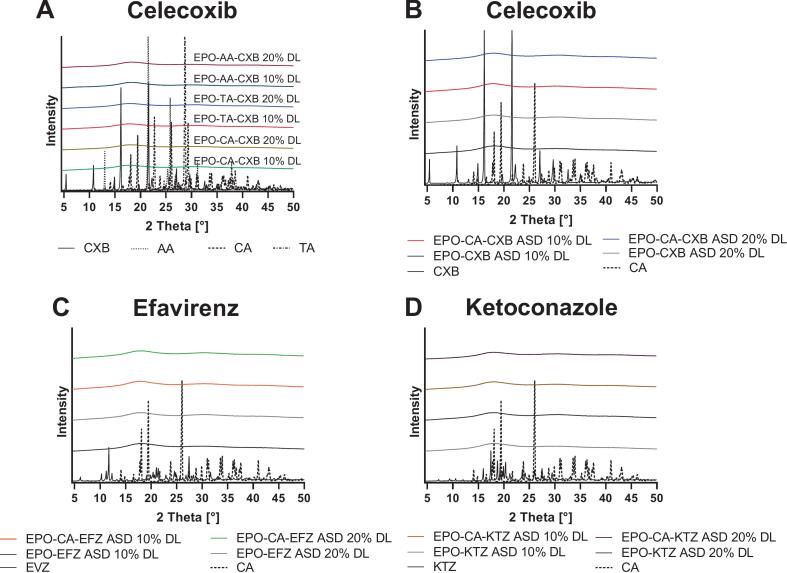
XRPD patterns of neat APIs and organic acids (AA, CA, TA) and corresponding EPO–acid ASDs. (A) CXB-containing ASDs with different acidic coformers (AA, CA, TA) at 10% and 20% drug load (DL); panels (B—D) show ASDs with CA only, comparing 10% and 20% DL for CXB (B), EFZ (C), and KTZ (D), each with and without the acidic conformer. Representative spectra are shown (*n* = 3).

As shown in Fig. 1, all neat APIs and pH modifiers displayed sharp Bragg reflections characteristic of their crystalline state, which were entirely absent in the processed ASD samples. Freshly prepared formulations exhibited only broad, diffuse halos throughout the measured 2θ range, indicating the complete transition to an amorphous structure.

#### 3.1.2 DSC – impact of acid type on ionic interactions within EPO-CXB-Based ASDs

**Fig. 2:**
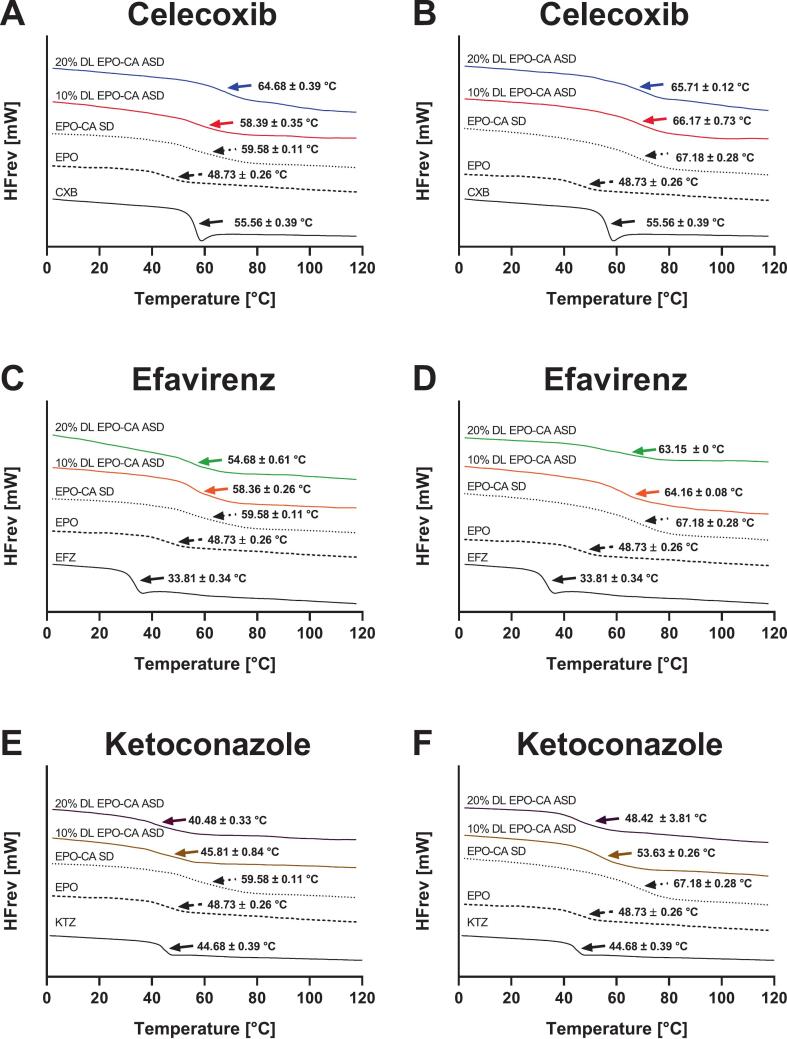
TOPEM DSC thermograms of neat drugs, neat EPO, EPO–CA blends, and corresponding ASDs at 10 and 20% DL during the first and second heat cycles. (A, B) CXB; (C, D) EFZ; (E, F) KTZ. Data are presented as mean ± standard deviation (SD) of three independent measurements (n = 3).

The glass transition temperature (T_g_) of neat EPO was 48.73 ± 0.26 °C, while amorphous CXB exhibited a T_g_ of 55.56 ± 0.39 °C, in good agreement with literature data (Kayser et al., 2022). A second heating cycle was not evaluated for these two compounds or for the other crystalline drugs, as no structural reorganization was expected in the absence of acid–polymer interactions. Incorporation of organic acids induced pronounced and acid-dependent changes in T_g_ (Fig. 2; detailed values are summarized in Table S3, Supplementary material). Adipic acid reduced T_g_ relative to neat EPO (37.59 ± 0.39 °C, second cycle), consistent with a plasticizing effect and increased chain mobility. In contrast, citric acid markedly increased T_g_, with the EPO-CA system exhibiting a T_g_ of 67.18 ± 0.28 °C in the second heating cycle, corresponding to a shift of approximately +18 °C relative to neat EPO. Tartaric acid resulted in an even more pronounced T_g_ increase, with values exceeding 90 °C in the pre-extruded EPO-TA blend. To evaluate whether these T_g_ shifts reflect deviations from ideal mixing behavior, theoretical T_g_ values were calculated using the Gordon–Taylor equation based on the T_g_ values of the neat components. The experimentally determined T_g_ values were in close agreement with the calculated values across all investigated systems, indicating the formation of homogeneous amorphous matrices without evidence of macroscopic phase separation. The change in T_g_ between the first and second heating cycles (ΔT_g_) was system-dependent. AA-based systems showed only minor shifts (ΔTg = 2 °C), whereas CA-containing formulations exhibited moderate increases (ΔT_g_ = 7–8 °C). The largest Δ T_g_ was observed for selected TA-containing ASDs, particularly for the EPO-TA-CXB system at 10% DL, where T_g_ increased by more than 20 °C upon reheating. These observations indicate differences in the equilibration behavior of the polymer–acid systems during the first heating cycle. While drug incorporation at 10% and 20% DL influenced the absolute T_g_ values of the systems, it did not alter the qualitative T_g_ hierarchy established by the respective acid type, indicating that matrix formation is primarily governed by polymer–acid interactions. In several formulations prepared solely via VCM, two T_g_ values were detected within a single heating cycle. Given the limited distributive mixing during VCM processing, this behavior is attributed to incomplete homogenization and diffusion-controlled equilibration within the sample. Upon storage, T_g_ values increased slightly while variability decreased, consistent with structural relaxation and progressive matrix homogenization. (Fig. S1, Supplementary material) FT-IR Spectroscopy.

**Fig. 3:**
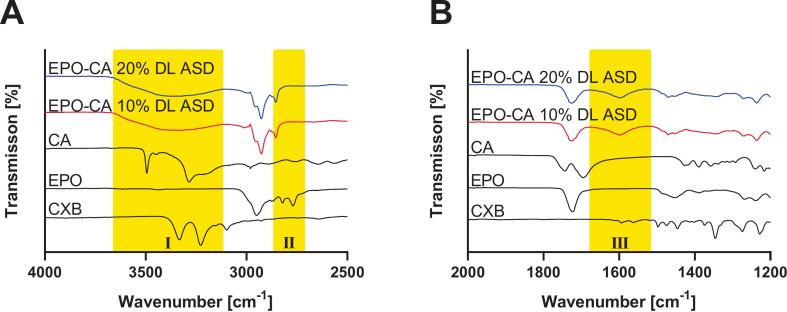
Fourier transform infrared (FT-IR) spectra of neat API, EPO, CA and EPO–CA–CXB ASDs containing 10 and 20% CXB (*w*/w): (A) 4000–2500 cm^−1^, (B) 2000–1200 cm^−1^. Representative spectra are shown (n = 3). Yellow regions (I-III) indicate the spectral areas discussed in the text. (For interpretation of the references to colour in this figure legend, the reader is referred to the web version of this article.)

To elucidate molecular interactions between the polymer matrix the acidic co-former CA and the API in this case exclusively CXB (Fig. 3; all other formulations can be found in the Supplementary material, Figs. S2 & S3), FT-IR spectra of the ASDs were compared with those of the corresponding neat components. EPO showed characteristic absorptions of the polymethacrylate backbone, including C—H stretching bands at 1450–1480 and 2950–2870 cm^−1^, C–O–C ester vibrations at 1150–1190, 1240 and 1270 cm^−1^, and a strong ester carbonyl band at approximately 1730 cm^−1^(Linares et al., 2019). The DMAE side chain exhibited two diagnostic bands at 2769 and 2821 cm^−1^; attributed to valence vibrations of non-ionized tertiary amine groups. CXB displayed NH stretching at 3332 and 3227 cm^−1^ and characteristic sulfonyl (SO_2_) stretching vibrations at 1345 cm^−1^ respectively; together with aromatic C=C stretching bands in the 1600–1500 cm^−1^ region (Pöstges et al., 2022). CA showed pronounced carbonyl stretching band of the protonated –COOH with a double peak at 1698 and 1743 cm^−1^; accompanied by a broad O—H stretching band extending from 2500 to 3500 cm^−1^. The FT-IR spectra of CA-containing ASDs revealed three distinctive changes relative to the corresponding physical mixtures. First; the disappearance of the carbonyl band of protonated carboxylic acids 1743 and 1698 cm^−1^ indicates deprotonation of CA upon processing. Second; the emergence of a new band at 1599 cm^−1^; matching the asymmetric stretch of –COO^−^; supports the formation of carboxylate species and thus ionic interactions. Third; the characteristic tertiary amine bands of EPO at 2770 and 2820 cm^−1^ showed reduced intensity; consistent with partial protonation of the DMAE groups (Fan et al., 2021). Additionally, all CA-containing ASDs exhibited a broadened absorption band at approximately 3339 cm^−1^, which can be assigned to N–H^+^ stretching vibrations of protonated tertiary amines. Collectively, these spectral changes provide strong evidence for acid–base interactions and salt formation between CA and the tertiary amine functionalities of EPO within the ASD matrix. All other investigated systems showed the same behavior in these three regions.

#### 3.1.3 ^1^H- and ^1^H—^1^H COSY NMR spectroscopy

**Fig. 4:**
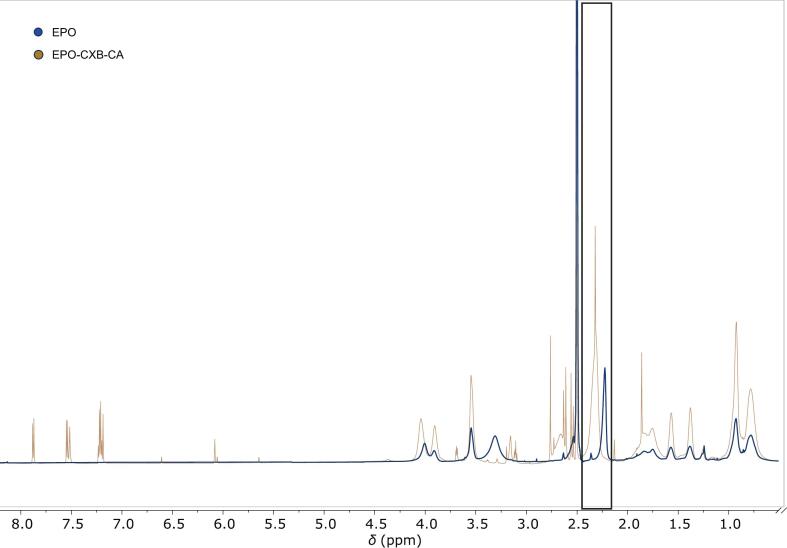
Superimposed ^1^H NMR spectra of neat EPO (500 MHz, DMSO‑*d*₆, 298 K) (blue) and the EPO-CA-CXB (700 MHz, DMSO‑*d*_6_, 298 K). amorphous solid dispersion (gold). The highlighted region shows the downfield shift and line broadening of the DMAE-associated signals upon addition of citric acid. (For interpretation of the references to colour in this figure legend, the reader is referred to the web version of this article.)

**Fig. 5:**
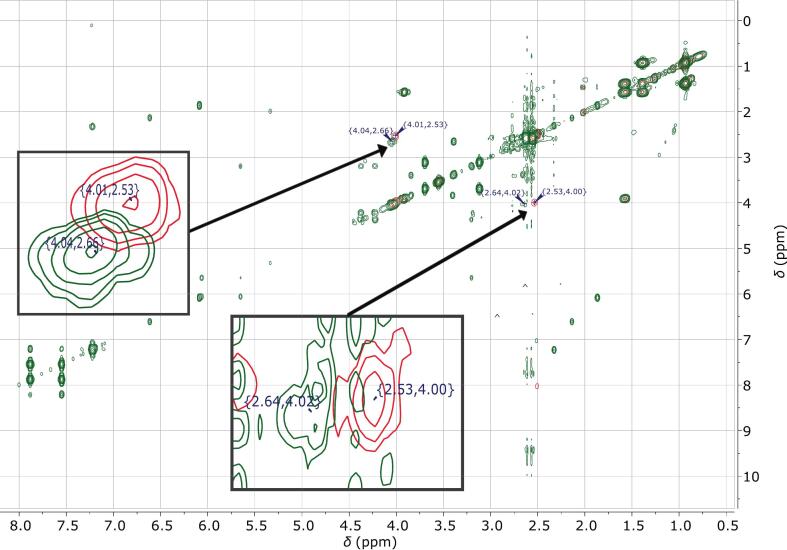
Superimposed ^1^H—^1^H COSY spectra of neat Eudragit EPO (red) and the EPO–CA–CXB ASD (green). Insets highlight enlarged cross-peak regions of the DMAE side chain showing a downfield shift after addition of CA, while all other cross-peaks remain unchanged. (For interpretation of the references to colour in this figure legend, the reader is referred to the web version of this article.)

The ^1^H NMR spectra (Fig. 4) showed that the structural integrity of EPO was preserved after ASD processing with CXB and CA. All characteristic polymer resonances remained clearly detectable and assignable in both the EPO-CXB and EPO-CA-CXB systems, indicating the absence of chemical degradation or covalent modification. As seen in Table S4 (Supplementary material) most EPO signals were highly conserved, with only minor chemical shift variations (≤ 0.03 ppm) for backbone-associated resonances, including the methoxy signal at 4.00 ppm (H5 & H8, for annotation see Supplementary Material, Fig. S4) and the aliphatic backbone region between 0.78 and 1.79 ppm (H1 & H3). In contrast, protons of the DMEA side chain exhibited localized changes upon addition of CA. The N–CH_2_ (H6) resonance, located at approximately 2.51 ppm in neat EPO and in the EPO-CXB system, shifted downfield and broadened to 2.71–2.60 ppm in the EPO-CXB-CA ASD. Similarly, the N–CH_3_ (H7) signal shifted from 2.20 ppm to 2.32 ppm and showed pronounced line broadening. The downfield displacement of these DMAE resonances becomes particularly apparent in the stacked spectra (Fig. 4), which were intensity-normalized to enable direct comparison of chemical shift positions. The combined downfield displacement and signal broadening indicate deshielding and reduced mobility of the tertiary amine environment and are consistent with protonation of the DMAE groups by CA Other polymer resonances, including the ester-associated methylene (H12) signal at approximately 4.00 ppm, remained essentially unaffected, confirming the site-specific character of these interactions. To support the structural assignments, ^1^H—^1^H COSY spectra were recorded for EPO/EPO-CXB in the presence and absence of CA (Fig. 5; supplementary material Figs. S5–8). The COSY cross-peak patterns were fully conserved across all systems and showed identical coupling networks, with no additional cross-peaks or altered coupling connectivity observed in the ASD system. Confirming that the polymer proton connectivity remained unchanged after drug incorporation and that the observed spectral changes reflected localized acid–base interactions rather than structural rearrangements.

### 3.2 Dissolution behavior under strongly non-sink conditions

Dissolution experiments were conducted under strongly non-sink conditions in PBS (pH 6.8) to probe the intrinsic supersaturation-generating capability of the EPO–acid matrices. Based on the crystalline solubilities of the APIs and the nominal dose concentration (200 μg/mL), the calculated sink index (SI) according to (Sun et al., 2016) confirmed strongly non-sink conditions for all compounds (SI ≤ 0.05). The relevant physicochemical properties and solubility values of the investigated APIs are summarized in Table 2. Under these stringent conditions, crystalline celecoxib exhibits a thermodynamic solubility below 1.5 μg/mL, providing a sensitive benchmark for assessing dissolution enhancement.

**Table 2:** Physicochemical properties and equilibrium/amorphous solubility of the investigated APIs.^a^

Compound	MW (g mol^−1^)	Log P	Tg (°C)	Tm (°C)	Thermodynamic solubility (μg/mL)^b^	Amorphous solubility (μg/mL)^b^	Sink index^c^
Celecoxib	381.4	3.5	55.6 ± 0.39	160	1.0 ± 0.05	23.7 ± 3.2	0.005
Efavirenz	315.7	4.7	33.8 ± 0.3	139	9.2 ± 0.09	32.4 ± 2.5	0.05
Ketoconazole	531.4	3.5	44.6 ± 0.39	150	5.8 ± 0.20	41.1 ± 0.3	0.03

a *n* = 3; errors indicate one standard deviation.

b in 0.05 M PO4-buffer at pH 6.8 and 37 °C.

c according to (Sun et al., 2016).

#### 3.2.1 Protonation degree governs dissolution behavior

**Fig. 6:**
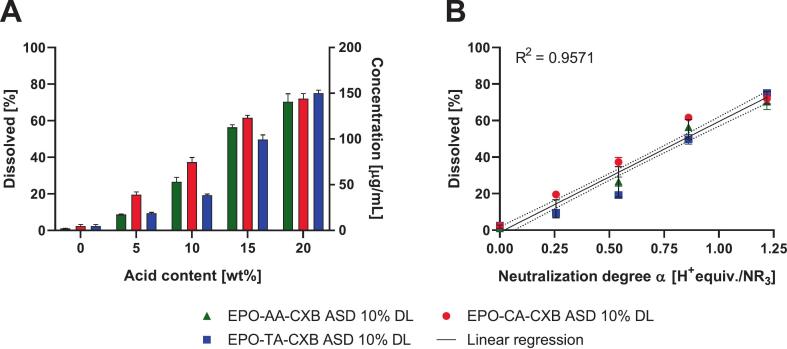
(A) CXB dissolved after 180 min from EPO-CXB ASDs with 10% DL and variation in acid content (neat, 5%, 10%, 15%, 20%). Non-sink dissolution study was conducted in 20 mL 0.05 M phosphate buffer at pH 6.8 (37 °C, 75 rpm paddle speed). (B) Correlation between neutralization degree α and CXB release from EPO-acid-CXB ASDs at 180 min. Symbols denote CA-, AA- and TA-based ASDs (mean ± SD, n = 3). The dotted line shows the linear regression fit with its 95% confidence interval.

Dissolution testing was conducted under monophasic, non-sink conditions to evaluate the pH-dependent release modulation induced by acidic excipients. The thermodynamic solubility of CXB in phosphate buffer (pH 6.8) was experimentally determined to be approximately 1 μg/mL, which is consistent with literature reports, describing extremely low solubility values typically below 1.5 μg/mL (Xie and Taylor, 2016). The resulting dissolution profiles exhibited a pronounced initial burst release in the first 15–20 min, followed by a quasi-stable plateau concentrations over the entire measurement period of 180 min. As seen in Fig. 6, increasing CA incorporation systematically increased the maintained CXB concentrations, reaching 163.67 ± 3.39 μg/mL at the highest acid content (20%), corresponding to more than a 100-fold enhancement relative to the crystalline solubility. Incorporation of other organic carboxylic acids (AA, TA) likewise induced an initial burst release followed by a rapid approach to a supersaturated plateau. Across all acids, the absolute plateau levels were markedly increased compared to crystalline CXB, but further increases in acid content did not necessarily translate into higher apparent solubility (Supplementary material, Fig. S9).

In summary, these data indicate that acidic pH-modifiers significantly enhance the apparent solubility of CXB, with citric acid showing a more pronounced concentration dependence, while structurally related dicarboxylic acids yield similarly high but less composition-sensitive supersaturation levels. To better rationalize these trends, CXB concentrations at 180 min were correlated with the calculated neutralization degree α, defined as the H^+^ equivalents per tertiary amine group (H^+^equiv./NR_3_), rather than with acid wt% alone. When plotted against α, all formulations followed an almost linear increase in apparent solubility, irrespective of the specific acid used, whereas no consistent relationship was obtained when release was plotted against acid wt% or acid pK_a1_ alone. This supports the view that the stoichiometric fraction of protonated EPO sites, rather than the absolute acid content, is the primary determinant of dissolution enhancement under the conditions studied. Expressing the system in terms of α therefore enables a unified correlation across different acid systems, reflecting the extent of polymer protonation rather than the nominal amount of acid present.

#### 3.2.2 Robustness across APIs, drug loadings, and biorelevant media

**Fig. 7:**
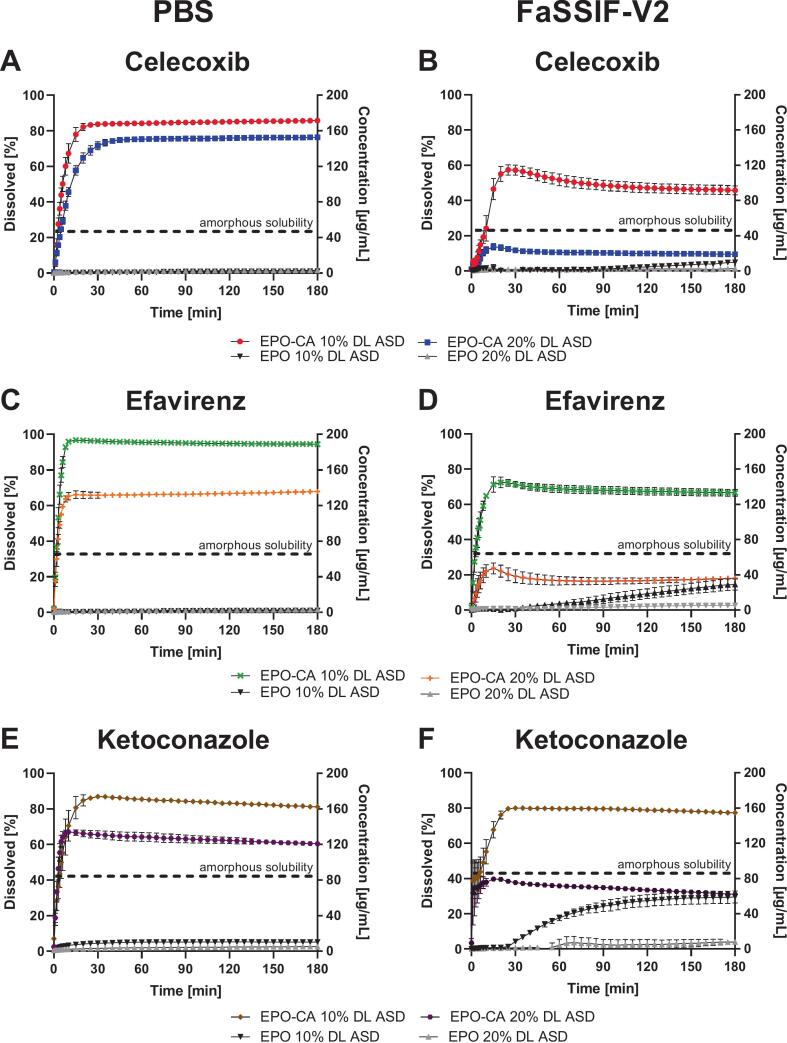
Non-sink dissolution of EPO-CA ASDs compared to EPO ASDs over 180 min at 75 rpm in 20 mL in PBS and FaSSIF-V2 (pH 6.8, c_max_: 200 μg/mL): CXB (A, B), EFZ (C, D) and KTZ (E, F). The dotted line indicates amorphous solubility. Values represent mean ± SD (n = 3).

In PBS, all three APIs exhibited pronounced and sustained supersaturation following release from the CA-modified ASDs. As seen in Fig. 7 both drug loadings led to a rapid increase in dissolved concentration, followed by a stable plateau throughout the 180 min experiment comparable to the preliminary tests before. For CXB, similar behavior to that observed in the previous section was obtained for the ASDs prepared via the HME/VCM process. The 10% DL reached a maximum concentration of 171.47 ± 2.71 μg/mL, (85% release), while the 20% DL achieved 152.73 ± 0.80 μg/mL (76%), corresponding to a 114-fold and 101-fold enhancement over the thermodynamic solubility, respectively. EFZ and KTZ showed comparable dissolution profiles over 180 min, both showing rapid supersaturation generation followed by sustained plateau concentration. At 10% DL EFZ an KTZ reached 188.93 ± 1.15 μg/mL and 159.73 ± 2.05 μg/mL, corresponding to approx. 19-fold and 31-fold supersaturation relative to their respective thermodynamic solubilities (10 μg/mL for EFZ and 5.13 μg/mL for KTZ) (Cristofoletti et al., 2013; Neuwirth et al., 2023). Increasing the drug loading to 20% slightly reduced the maintained concentrations but did not alter the overall dissolution behavior. Overall, the citric-acid-modified ASDs enabled robust generation and stabilization of supersaturation in PBS across all three APIs, with a consistently stronger effect observed at the lower drug loading. In FaSSIF-V2, all ASDs showed reduced absolute concentrations but still achieved clear supersaturation. For example, CXB reached 91.47 ± 5.15 μg/mL, while EFZ and KTZ likewise maintained supersaturated concentrations across both DL Compared with PBS, FaSSIF-V2 dissolution showed a delayed onset with a transient early maximum and slight decline, likely caused by bile salt–polymer interactions at the matrix–medium interface as increasing transmission in the beginning of the measurements indicated (Data not shown). Nevertheless, supersaturation was sustained across all APIs and both drug loadings, with the strongest effect at 10% DL. The higher buffer capacity (Bapat et al., 2023) of FaSSIF-V2 may additionally contribute to the delayed dissolution onset, Overall, these results demonstrate that the acid–polymer interactions governing stabilization in the EPO–CA system remain effective under biorelevant conditions.

#### 3.2.3 Kinetics of matrix erosion and phase behavior

**Fig. 8:**
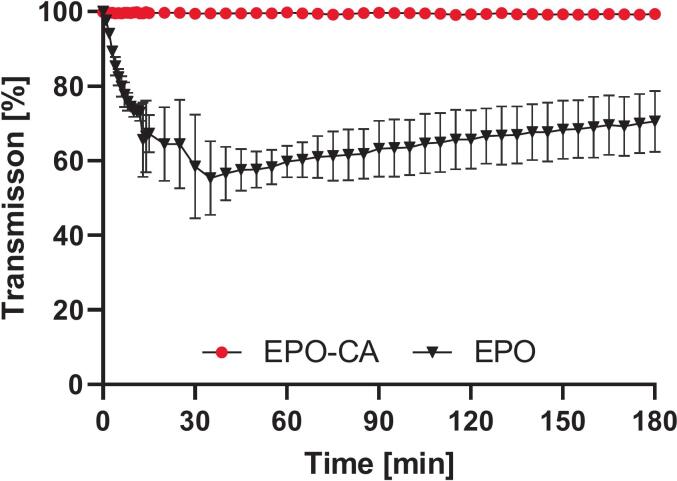
Transmission-based turbidity monitoring of placebo polymer systems during dissolution in PBS (pH 6.8, 37 °C) over 180 min to assess polymer-induced turbidity. Data represent mean ± SD (n = 3).

To assess whether the enhanced dissolution performance originates from polymeric dispersion or a more controlled matrix erosion, placebo systems containing neat EPO or EPO-CA were investigated using inline turbidity monitoring (Fig. 8). Neat EPO exhibited a characteristic transient increase in light scattering during the early stages of the experiment. This peak in turbidity suggests an incoherent disintegration of the polymer, where the matrix breaks down into temporary, undissolved aggregates upon contact with the medium before slowly solvating. In contrast, the EPO-CA systems showed no measurable increase in turbidity throughout the entire dissolution period. This absence of light scattering indicates a fundamental shift in the dissolution mechanism: the acid-modified polymer undergoes rapid, congruent erosion. In this process, the in-situ protonation of the tertiary amine groups at the solid-liquid interface allows the polymer chains to be solvated directly, effectively bypassing the formation of detectable dispersed phases. These observations suggest that the incorporation of acidic coformers does not promote the formation of persistent polymer dispersions. Instead, it facilitates a highly efficient surface erosion driven by the increased ionization of the alkaline polymethacrylate under near-neutral conditions. This mechanistic interpretation is further supported by complementary particle size measurements (Supplementary material, Fig. S11), which confirm the absence of larger particulate species in the acid-modified systems.

#### 3.2.4 Precipitation inhibition in solution

**Fig. 9:**
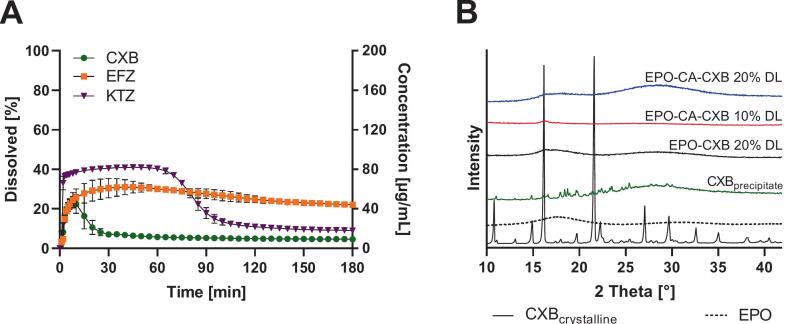
(A) Supersaturation spike experiment of CXB, EFZ and KTZ in 20 mL 0.05 M phosphate buffer at pH 6.8 (37 °C, 75 rpm paddle speed. All APIs show loss of supersaturation due to precipitation, with compound-specific precipitation kinetics. Values represent mean ± SD (n = 3) (B) XRPD patterns of the precipitate obtained from the CXB supersaturation experiment after 180 min (wet sample) compared with crystalline CXB reference.

The supersaturation spike experiment revealed pronounced precipitation of all investigated APIs under polymer-free conditions (Fig. 9). Following generation of the supersaturated state, dissolved concentrations decreased over time, indicating loss of supersaturation through precipitation. The precipitation kinetics differed between the compounds, with CXB showing the fastest decline, whereas EFZ and KTZ maintained elevated dissolved concentrations for longer periods before gradually approaching lower steady-state levels.

To determine the solid-state nature of the precipitated material, the solid phase recovered from the CXB supersaturation experiment after 180 min was analyzed by XRPD (Fig. 9B). The diffractogram showed several reflections corresponding to crystalline CXB, confirming that precipitation from supersaturated solution results in partial drug recrystallization in the absence of polymer. Additional reflections not attributable to CXB were observed and likely originate from phosphate buffer salts present in the PBS medium. In contrast, solids recovered from ASD dissolution experiments exhibited predominantly broad halos, indicating that precipitation in the presence of the polymer matrix resulted largely in amorphous material. Only a weak shoulder in the CXB region (around 2θ ≈ 16–17°) was observed for the EPO-CA–CXB 10% DL sample. However, this formulation yielded only a small amount of recovered material, and the relative intensity of this feature should therefore be interpreted cautiously. XRPD measurements performed after 24 h drying (data not shown) did not show a substantial increase in CXB reflections for the ASD-derived solids, indicating that no pronounced crystallization occurred during solvent removal. Taken together, these results demonstrate that while CXB readily recrystallizes following precipitation from supersaturated solution, precipitation occurring during ASD dissolution yields predominantly amorphous solids. This behavior is consistent with polymer-mediated inhibition of crystalline drug precipitation, which contributes to the sustained supersaturation observed in the dissolution experiments.

#### 3.2.5 Performance under physiologically relevant pH conditions

**Fig. 10:**
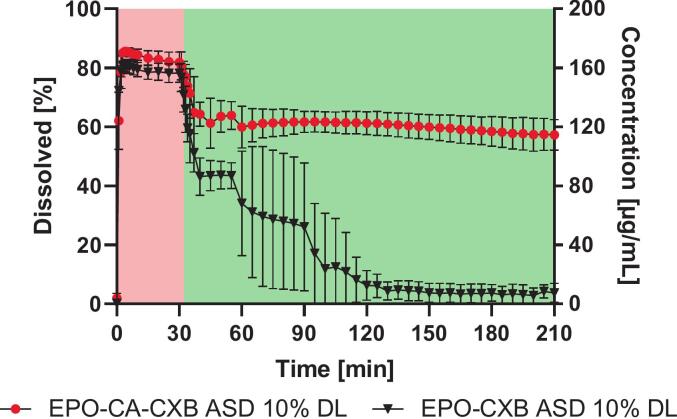
pH-shift dissolution of EPO-CA-CXB ASDs compared to EPO-CXB ASDs during a gastric-to-intestinal transition at 75 rpm in 20 mL dissolution medium (c_max_: 200 μg/mL). The red shaded region indicates the acidic stage (pH 1.2), followed by the intestinal stage at pH 6.8 (green region). Values represent mean ± SD (n = 3). (For interpretation of the references to colour in this figure legend, the reader is referred to the web version of this article.)

To evaluate formulation performance under physiologically relevant conditions, a pH-shift dissolution experiment mimicking gastric-to-intestinal transit was performed (Fig. 10). During the initial acidic stage (pH 1.2), both formulations showed very similar dissolution levels, reaching comparable drug concentrations within the first 30 min. This behavior is consistent with the dissolution profiles observed in the preceding experiments. After the transition to pH 6.8, the dissolution profiles began to diverge. The EPO-CXB ASD showed a pronounced decline in dissolved drug concentration, decreasing to near baseline levels within approximately 90–120 min. In contrast, the EPO-CA-CXB ASD maintained substantially higher dissolution levels after the pH shift and retained approximately 60% of the initially dissolved drug after 210 min. The moderate decline observed after the shift likely reflects the abrupt buffering conditions during the pH transition. Addition of the concentrated phosphate buffer solution, which contains NaOH for pH adjustment, may transiently generate locally alkaline microenvironments with short-lived pH spikes, under which rapid neutralization of the polymer can occur and precipitation may be promoted in systems lacking sufficient residual protonation. Overall, the comparable dissolution levels under acidic conditions and the pronounced differences after the pH shift support the interpretation that supersaturation stabilization is governed primarily by the protonation state and solubility of the polymer matrix rather than by a direct solubilizing effect of the incorporated acid.

### 3.3 Stability

#### 3.3.1 Acid Retention after processing

HPLC analysis was used to quantify the amount of organic acids remaining after thermal processing. The processed ASD samples showed high acid retention for all investigated acids. Citric acid exhibited a retention of 95.6 ± 3.5%, while tartaric acid and adipic acid showed retentions of 97.7 ± 3.3% and 99.2 ± 0.2% (*n* = 3), respectively. These results indicate that only minimal acid loss occurred during processing and suggest that the targeted stoichiometric acid–base ratios introduced during formulation were largely preserved.

#### 3.3.2 Stability under long-term and accelerated storage conditions

For CXB ASDs (10 and 20% *w*/w), XRPD patterns remained fully amorphous after storage at 25 °C/40% RH and 40 °C/75% RH, and dissolution profiles were unchanged for all three acids, indicating absence of recrystallization and preserved release performance. For EFZ- and KTZ-based ASDs, accelerated storage at 40 °C/75% RH likewise showed no detectable crystallinity in XRPD and no relevant changes in drug release compared with initial profiles. All Figures can be found in the supplementary Data (Supplementary material Figs. S12–15).

## 4 Discussion

In the present work, we demonstrate that incorporating organic acids into EPO-based ASDs reorganizes the polymer matrix into an ionically crosslinked polymer–acid network that controls thermal behavior, physical stability and dissolution performance. XRPD confirmed complete amorphization of all three APIs in all EPO-acid-API systems, irrespective of whether citric, adipic or tartaric acid was used. However, these systems deviate from classical binary polymer-API dispersions: instead of a T_g_ located between those of EPO and the drug, the ternary formulations exhibit a new, higher T_g_ governed by polymer–acid interactions, indicating restricted chain mobility (Wu et al., 2018). The distinct T_g_ hierarchy between the acid systems; together with the pronounced T_g_ increases upon acid incorporation; supports specific polymer–acid interactions beyond conventional plasticization. This behavior is consistent with the protonation extent of the DMAE side chains of EPO by the respective acids and the formation of transient ionic crosslinks within the polymer network (Guzmán et al., 2012; Hunley et al., 2010). DSC on reheating, i.e., second heating cycle, revealed a further T_g_ increase, implying that a fraction of tertiary amine groups remained unprotonated after the first processing and became accessible only when additional molecular mobility was provided. Notably, the change in Tg between the first and second heating cycles followed the order AA < CA < TA, indicating differences in the equilibration kinetics of polymer–acid interactions. While the absolute Tg reflects the strength of the resulting polymer–acid network, ΔTg indicates the kinetics by which this state is established. The largest ΔTg values were observed for selected TA-containing systems and were in some cases influenced by drug loading, suggesting slower equilibration within the amorphous matrix. Similar acid- and history-dependent ionization of EPO under harsh conditions has been reported (Linares et al., 2019). Protonation was therefore dictated not only by acid content but also by kinetic accessibility of protonation sites; in line with process-dependent proton transfer described for amorphous drug–polymer salts (Neusaenger et al., 2023). Stability data support this dynamic picture. Under normal storage; T_g_ increased over time; consistent with a gradual shift toward a more extensively protonated; thermodynamically favored salt-like state; analogous to the evolution observed in amorphous drug–polymer salts (Neusaenger et al., 2023). Under accelerated thermal stress (second heating cycle); T_g_ partially decreased; indicating that the protonation state can relax when mobility is increased and that a reversible equilibrium is established (Linares et al., 2019). Taken together; the polymer–acid network behaves as a metastable; adaptive system whose properties depend strongly on processing conditions; thermal history and storage environment. FTIR provided molecular-level evidence for this mechanism. A new band at 3339 cm^−1^; attributed to protonated tertiary amines of the DMAE side chains; together with attenuation of the 2770 and 2820 cm^−1^ bands from non-protonated amines; indicates progressive consumption of free amine functionalities. Concurrently; a strong band at 1599 cm^−1^; characteristic of asymmetric carboxylate stretching; confirms deprotonation of the organic acids and ammonium–carboxylate pair formation; as also reported for EPO-acidic drug complexes and acid-treated derivatives (Linares et al., 2019; Quinteros et al., 2008). Complementary ^1^H NMR measurements of neat EPO, EPO-CXB and EPO-CXB-CA corroborated localized protonation: methylene protons adjacent to the tertiary amine (H6) and dimethylamino protons (H7) shifted downfield and broadened, whereas ester-adjacent methylene and backbone signals remained essentially unchanged. This pattern indicates that interactions are confined to the cationic DMAE segment rather than causing global conformational changes. Overall, NMR and FTIR support an internal polymer–acid salt-like structure with localized EPO protonation, explaining the T_g_ increase and enhanced physical stability observed by DSC and XRPD (Hunley et al., 2010; Neusaenger et al., 2024; Neusaenger et al., 2023) and matching mechanistic findings for saccharin-modified EPO ternary systems (Higashi et al., 2016; Ueda et al., 2018b).

HPLC analysis confirmed that the majority of the incorporated organic acids remained in the formulation after thermal processing, indicating minimal acid loss and supporting the assumed stoichiometric acid–base interactions within the polymer matrix. Given the potential thermal sensitivity of CA and TA, verification of acid retention was particularly important and confirmed only minor losses under the applied processing conditions. From a manufacturing perspective, the choice of acids also reflects practical constraints of the intended processing route. Citric and tartaric acid exhibit limited thermal stability and may therefore be more suitable for solvent-based preparation routes such as spray drying or solvent evaporation, or for melt processing under very short residence times. In contrast, adipic acid represents a thermally more robust diacid and is therefore particularly attractive for melt-based processing such as HME or VCM. To assess whether the observed ionization-driven behavior is restricted to melt processing, a solvent-based preparation of the polymer–acid system was additionally investigated (Supplementary material, Fig. S10). Comparable qualitative behavior was observed, indicating that the underlying polymer–acid interactions are not unique to thermal processing. Related strategies in which interacting additives are introduced prior to thermal processing to generate polyelectrolyte-like matrices have previously been reported by (Ditzinger et al., 2019) highlighting the broader applicability of ionic structuring approaches in ASD design. In contrast, the present study demonstrates that comparable ionic structuring of the polymer matrix can also be achieved directly during thermal processing when residence times are appropriately Accordingly, the present work should be viewed as a proof-of-concept demonstrating protonation-controlled polymer structuring under melt-processing conditions rather than an optimized manufacturing process.

Beyond structural and thermal effects, the ionic network strongly affected dissolution under non-sink conditions The observed release behavior indicates that drug release from the ASD is primarily governed by protonation-dependent polymer solubilization rather than by simple matrix erosion. Turbidity monitoring and laser diffraction measurements confirmed progressive matrix disintegration during dissolution, indicating that polymer dissolution contributes to drug release. However, the sustained supersaturation profiles observed for the acid-modified systems suggest that dissolution alone cannot explain the observed behavior. The observed release profiles align with the principles of ionization-driven polymer solubilization and disentanglement (Bapat et al., 2024; Hiew et al., 2022). For Ketoconazole, the acidic microenvironment likely maintains a cationic state at the interface, promoting electrostatic repulsion between the drug and the EPO matrix, which facilitates matrix erosion. Conversely, for the neutral APIs CXB and EFZ, the ionic network compensates for the lack of intrinsic drug–polymer interactions by providing the necessary protonation to drive these solubilization processes. In EPO–CXB ASDs, increasing CA content systematically enhanced both dissolution rate and maximum supersaturation. The use of different acids produced distinct profiles reflecting their proton-donating capacity and dissociation behavior. The three acids differ in their intrinsic acidity, with first dissociation constants of approximately pK_a₁_ = 2.98 (TA), 3.13 (CA) and 4.43 (AA). Under the sub-stoichiometric conditions employed here (acid equivalents ≤ basic EPO equivalents), proton transfer to EPO is effectively governed by this first deprotonation step; the higher dissociation steps of the polyprotic acids (pK_a₂_, for CA and TA; pK_a₃_ for CA) are only partially accessed in the glassy, low-mobility matrix and therefore contribute little to the overall charge balance. Consequently, pK_a₁_ is the relevant descriptor of acid strength in this system. CA, as a triprotic acid, produced a rapid initial concentration increase followed by a gradual rise in supersaturation, whereas AA- and TA-acid systems rapidly reached an early plateau. At a given mass fraction, the three available protons of CA allow higher neutralization degrees of EPO than AA or TA, which can result in slightly more pronounced ionic modulation of the polymer network. However, the overall dissolution behavior of all three acid-modified systems was broadly similar: AA- and TA-based ASDs also generated high and sustained supersaturation, and differences between acids were modest and best rationalized by the achieved neutralization degree at a given composition rather than by an intrinsic superiority of a particular acid. Consistently, CXB release at 180 min correlated strongly with the calculated neutralization degree α whereas plotting release against acid pK_a1_ did not yield a meaningful relationship (data not shown). While acid wt% produced monotonic trends within individual acid series, α provided a unified descriptor across acids, indicating that the stoichiometrically defined fraction of protonated EPO sites is more predictive for dissolution performance than nominal acid concentration or pK_a_ alone. Control experiments further clarified the origin of the observed supersaturation behavior. Supersaturation spike experiments performed in the absence of polymer showed a rapid loss of supersaturation, demonstrating that the drugs readily precipitate from supersaturated solution under these conditions. In contrast, the presence of EPO in the ASDs significantly prolonged supersaturation, indicating polymer-mediated precipitation inhibition. XRPD analysis of solids recovered after dissolution provided further insight into the precipitation process. Precipitates obtained from spike experiments in buffer in the absence of polymer exhibited partially crystalline diffraction patterns characteristic of CXB. In contrast, solids recovered from ASD dissolution experiments were dominated by broad amorphous halos without distinct CXB reflections. This comparison indicates that the polymer matrix suppresses direct crystalline precipitation pathways and instead promotes the formation of amorphous precipitates during dissolution (Ueda et al., 2025). These structural and interfacial effects translated into dissolution performance in PBS and FaSSIF-V2. Although bile salts and phospholipids and buffer-capacity effects reduced absolute drug release (c_max_) and delayed supersaturation onset (Bapat et al., 2023; Schlauersbach et al., 2022a), all three acid-modified EPO systems generated and sustained marked supersaturation for the three APIs, with only subtle differences in extent and duration that reflected formulation-specific neutralization degrees (Fig. 6).This strong effect of acid-modified EPO on dissolution and supersaturation mirrors previous findings with saccharin-based ternary systems (Ueda et al., 2018a). Physiological relevance was further addressed using pH-shift dissolution experiments. Both formulations reached comparable dissolution levels under acidic conditions; whereas pronounced differences emerged only after transition to intestinal pH. This behavior indicates that the primary benefit of acid incorporation is the preservation of polymer protonation and solubility under near-neutral intestinal conditions rather than enhanced dissolution in the gastric stage. In practical dosage forms; gastric exposure could also be bypassed by using enteric capsules or delayed-release formulations that release the ASD directly under intestinal conditions (Monschke et al., 2021).

Overall, this study shows that organic acids play a dual, mechanistically coupled role in EPO-based ASDs. They induce a dynamically protonated polymer network that raises T_g_ and restricts molecular mobility, improving resistance to recrystallization and aligning with the broader concept of amorphous drug-polymer salts as high-T_g_, low-mobility systems (Neusaenger et al., 2023). On the other hand; they modulate drug release and supersaturation through controlled ionic interactions and microenvironmental pH effects at the dissolving interface; as established for pH-modified solid dosage forms and EPO-based ternary dispersions. In the broader ASD landscape; clinically established ASD polymers are typically pH-independent or enteric systems such as HPMCAS or PVP-based polymers (Moseson et al., 2024). In contrast, alkaline polymethacrylates such as EPO remain underutilized due to their pronounced pH-dependent solubility across the GI tract. This creates a clear formulation gap: EPO offers strong ionic interaction potential and efficient amorphization, but its limited solubility under intestinal conditions restricts consistent performance. The present work addresses this gap by demonstrating a formulation strategy that controls polymer protonation independently of drug acidity, thereby enabling EPO-based ASDs to function under near-neutral and biorelevant conditions. The fact that the protonation state evolves progressively and remains partially reversible means that these systems are best viewed as adaptive molecular assemblies whose properties are determined by processing conditions, thermal history and surrounding environment. In this way, pH-modifiers effectively unlock alkaline Polymers as versatile carriers for poorly soluble APIs of diverse ionization states under physiological conditions, providing a mechanistic basis for the rational design of ionically structured ASDs with tunable stability and dissolution behavior. Importantly, this mechanistic framework is not restricted to EPO or the organic acids investigated here, but is expected to be transferable to other alkaline polymethacrylates and pH-modified polymer systems. As such, the present findings establish a general design principle for enabling alkaline polymers as functional ASD carriers under physiological conditions, independent of specific API chemistry.

## 5 Conclusion

This work demonstrates that incorporating organic acids into EPO-based ASDs transforms EPO from a simple carrier into a dynamically protonated, ionically crosslinked polymer–acid network that controls both solid-state properties and dissolution performance. By combining XRPD, DSC, FTIR, and ^1^H NMR, we show that localized protonation of the DMAE side chains generates a salt-like matrix with increased T_g_ and reduced molecular mobility, while remaining adaptable to processing and storage conditions. These structural changes translate into pronounced improvements in dissolution rate and supersaturation in PBS and FaSSIF-V2, driven by a shift toward congruent matrix erosion. With citric acid in particular enabling sustained supersaturation across three poorly soluble drugs. Importantly, dissolution behavior was primarily governed by the effective degree of polymer protonation rather than by acid content alone, highlighting protonation-controlled polymer behavior as the key design parameter. Overall, organic-acid-modified EPO ASDs emerge as adaptive molecular assemblies whose protonation state and performance can be tuned via acid type, acid content and thermal history, providing a mechanistic basis for the rational design of ionically modulated ASDs to enhance the oral bioavailability of poorly soluble APIs. Placed in the broader context of ionizable ASD carriers, these findings further support the emerging concept that controlled ionic interactions can modulate polymer matrix behavior in amorphous formulations. In particular, the present results highlight the potential of alkaline polymers as adaptable carriers whose protonation state, and thus dissolution performance can be deliberately adjusted through acid–base interactions.

## Declaration of generative AI and AI-assisted technologies in the manuscript preparation process

During the preparation of this work, the author used BioRender AI, ChatGPT and Gemini to assist with figure design, language refinement, abstract optimization, and improving the clarity of the title and highlights. After using these tools, the author reviewed and edited the content as needed and takes full responsibility for the content of the published article.

## CRediT authorship contribution statement

**Lukas Mild:** Writing – original draft, Visualization, Validation, Methodology, Investigation, Formal analysis, Data curation, Conceptualization. **Alicia Stakemeier:** Writing – review & editing, Visualization, Methodology, Investigation. **My Ngoc Nguyen Thi:** Visualization, Methodology, Investigation. **Karl G. Wagner:** Writing – review & editing, Supervision, Resources, Project administration, Conceptualization.

## Funding

This research did not receive any specific grant from funding agencies in the public, commercial, or not-for-profit sectors.

## Declaration of competing interest

The authors declare that they have no known competing financial interests or personal relationships that could have appeared to influence the work reported in this paper.

## Data Availability

Data will be made available on request.
