# Supplementary material for: Unlocking the potential of alkaline polymethacrylates via ionic crosslinking in amorphous solid dispersions
Source: Int J Pharm X. 2026 Apr 1;11:100531. doi: 10.1016/j.ijpx.2026.100531 (PMC13092032; doi:10.1016/j.ijpx.2026.100531)
Supplement: Supplementary file 1 — Tables and Figures [file mmc1.pdf]

Appendix A. Supplementary Data

Tables

A 1 Calculation of neutralization degree

Table S1: Calculation of the DMAE group content in Eudragit® E PO based on the alkaline value (AV).

**Parameter**	**Symbol / Equation**	**Value**	**Unit**
Alkaline value (AV)[a] of polymer	AV	180	mg KOH /g polymer
Conversion to grams	AV	0.180	g KOH/g polymer
Molar mass of KOH	M_{KOH}	56.11	g/mol
Moles of KOH per g polymer	$n = AV / M_{KOH}$	3.2×10^{-3}	Mol/g
DMAE groups per g polymer (1:1 equivalence with KOH)	n_{DMAE}	3.2	Mmol/g
DMAE groups per repeat unit (copolymer ratio 2:1:1)	—	≈ 0.40	mol per repeat unit

[a] amount of KOH required to neutralize the basic groups in the polymer (mg KOH/g polymer).

A 2 Protonation Degree Governs Dissolution Behavior

Table S2: Composition and molar ratios of EPO-CA-CXB ASDs (per 100 g total mass, DL = 10%).

**CA (%)**	**EPO (g)**	**n_{CXB} (mol)**	**n_{DMAE} (mol)**	**n_{CA} (mol)**	**CXB:DMAE:CA**	**$H^+_{equiv.}/NR_3$**
5 %	85.00	0.02622	0.30535	0.02603	1 : 11.645 : 0.993	0.256
10 %	80.00	0.02622	0.28730	0.05201	1 : 10.955 : 1.983	0.542
15 %	75.00	0.02622	0.26925	0.07812	1 : 10.273 : 2.979	0.868
20 %	70.00	0.02622	0.25146	0.10416	1 : 9.590 : 3.974	1.243

A 3 Impact of Acid Type on Ionic Interactions within EPO-CXB-Based ASDs

Table S3: Effect of acidic co-formers and drug load on the glass transition behavior of EPO-based ASDs.

**Name**	**First run (° C)**	**Second run (° C)**
EPO	48.73 ± 0.2	-
CXB	55.56 ± 0.39	-
EFZ	33.81 ± 0.34	-
KTZ	44.68 ± 0.39	-
EPO-AA	37.59 ± 0.39	39.48 ± 0.22
EPO-CA	59.58 ± 0.11	67.18 ± 0.28
EPO-TA	90.48 ± 0.36*<br>53.65 ± 0.19	90.81 ± 0.38*
EPO-AA-CXB 10% DL	39.64 ± 0.42	42.50 ± 0.29
EPO-AA-CXB 20% DL	42.37 ± 0.22	48.59 ± 0.19
EPO-CA-CXB 10% DL	58.39 ± 0.35	64.68 ± 0.39
EPO-CA-CXB 20% DL	58.39 ± 0.35	65.71 ± 0.12
EPO-TA-CXB 10% DL	55.15 ± 2.17	77.52 ± 2.13
EPO-TA-CXB 20% DL	83.91 ± 0.80	83.48 ± 0.33
EPO-CA-EFZ 10% DL	58.36 ± 0.26	64.16 ± 0.08
EPO-CA-EFZ 20% DL	54.83 ± 0.61	63.15 ± 0.02
EPO-CA-KTZ 10% DL	45.81 ± 0.84	53.63 ± 0.26
EPO-CA-KTZ 20% DL	40.48 ± 0.33	48.42 ± 3.81

* The value marked with an asterisk corresponds to the dominant, experimentally observable T_g. The second value represents a minor inflection within the thermogram.

A 4 ^{1}H- and ^{1}H-^{1}H COSY NMR Spectroscopy

Table S4: ^{1}H-NMR Chemical shifts of EPO in the presence of CXB and CA measured in DMSO-d_6.

**Position**	**EPO**	**EPO-CXB**	**EPO-CXB-CA**
1	1.79 br d	1.79 br s	1.79 br s
2	-	-	-
3	0.78 br s	0.78 br s	0.78 br s
4	-	-	-
5	4.01 br s	3.99 br s	4.04 br s
6	~2.54 m[a]	~2.51[a]	2.71-2.60 br s[b]
7	2.23 s	2.20 s	2.32 br s[c]
8	3.91 br s	3.91 br s	3.90 br s
9	1.57 br s	1.57 br s	1.56 br s
10	1.38 br s	1.38 br s	1.37 br s
11	0.93 br s	0.93 br s	0.92 br s
12	3.55 s	3.55 s	3.54 s

[a] Overlaps with solvent signal (DMSO-d_6 = 2.50 ppm)

[b] Overlaps with signals of CA (H3)

[c] Overlaps with signal of CXB (H1)

Figures

A 5 DSC – Impact of Acid Type on Ionic Interactions within EPO-CXB-Based ASDs

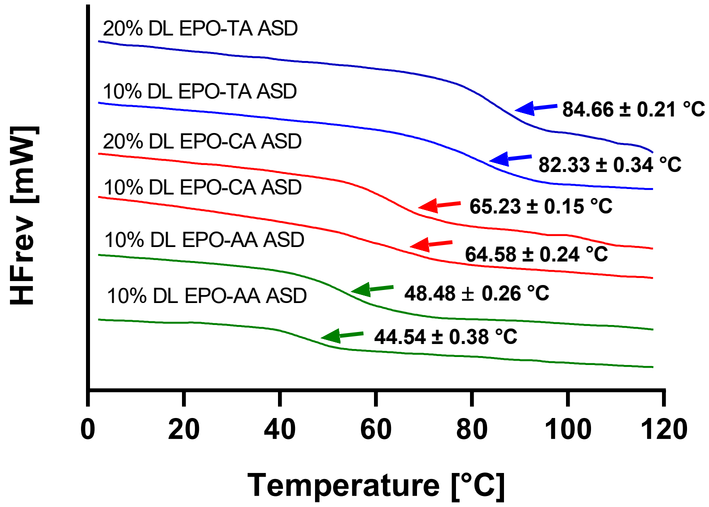

Figure S1: DSC thermograms of EPO-acid-CXB ASDs after four weeks of storage. Thermograms are shown for systems containing AA, CA, and TA at 10 and 20 % DL during the first heating cycles. Arrows indicate the observed T_g transitions. Data are presented as mean ± standard deviation (SD) of three independent measurements (n = 3).

A 6 FT-IR Spectroscopy

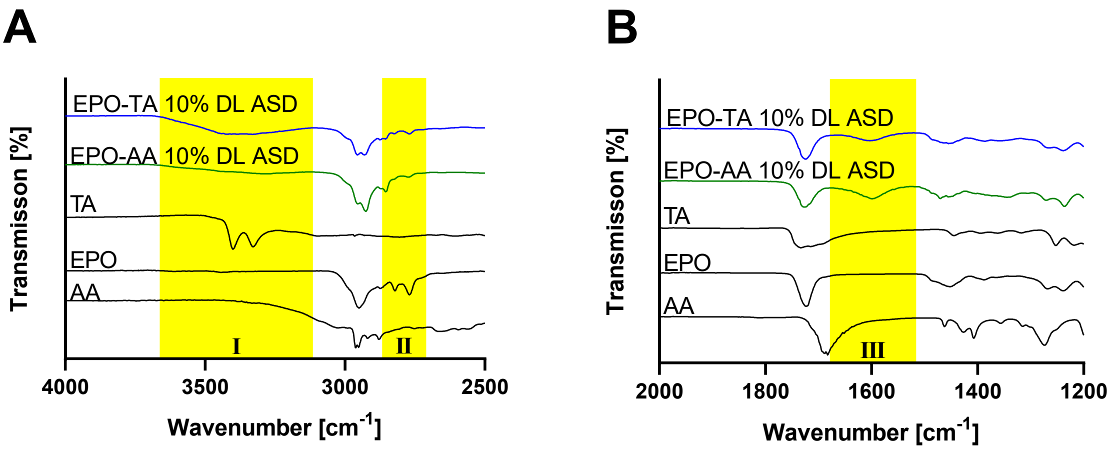

Figure S2: FT-IR spectra of neat CXB, EPO, AA, TA and corresponding EPO-acid-CXB ASDs containing 10% CXB (w/w): Yellow regions indicate the spectral areas discussed in the text.

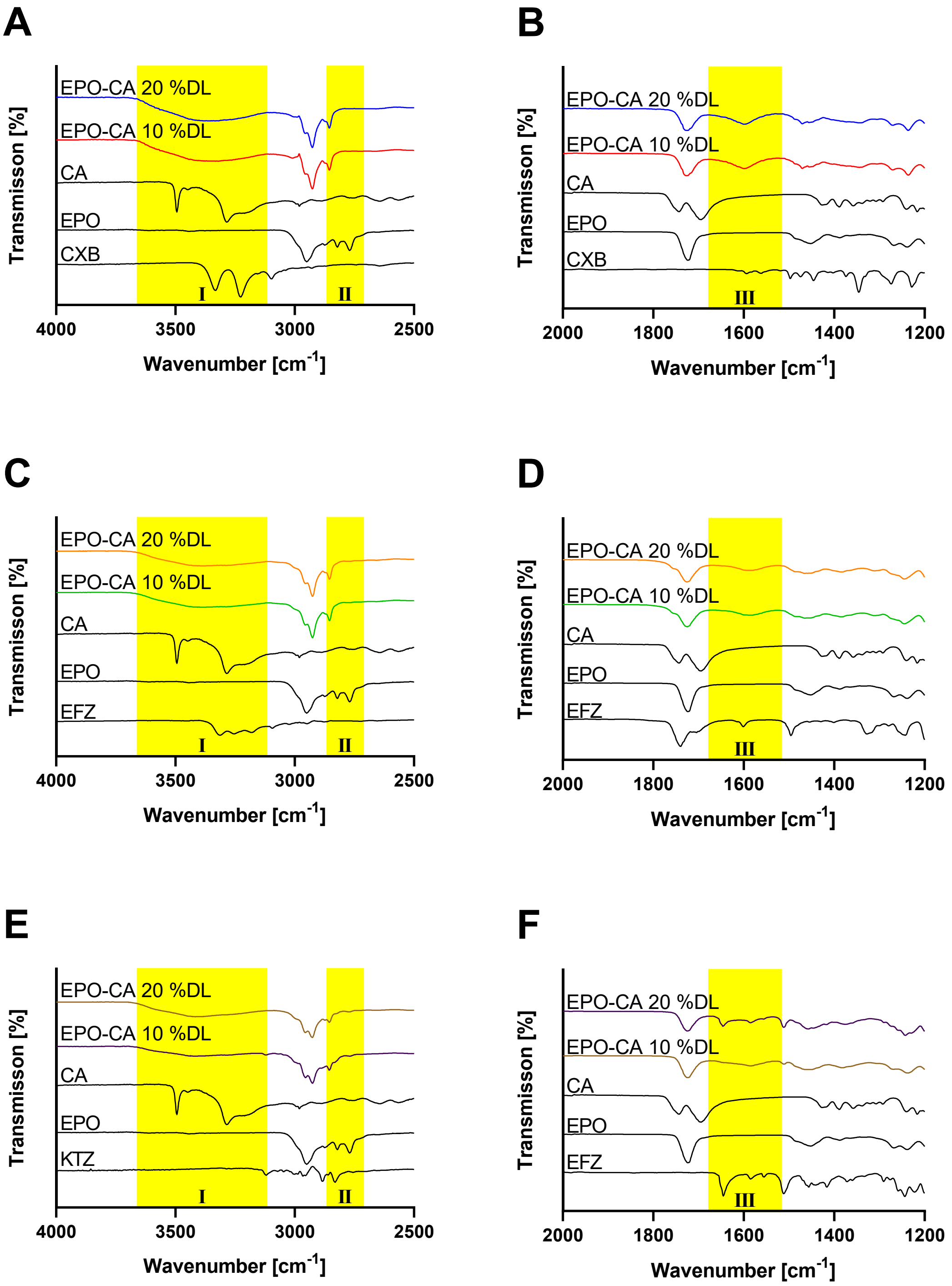

Figure S3: FT-IR spectra of neat API, EPO, CA and EPO-CA-CXB/EVZ/KTZ ASDs containing 10 and 20% CXB (w/w): (A/C/E) 4000–2500 cm⁻¹, (B/D/F) 2000–1200 cm⁻¹.

A 7 ^{1}H and ^{1}H-^{1}H COSY NMR Spectroscopy

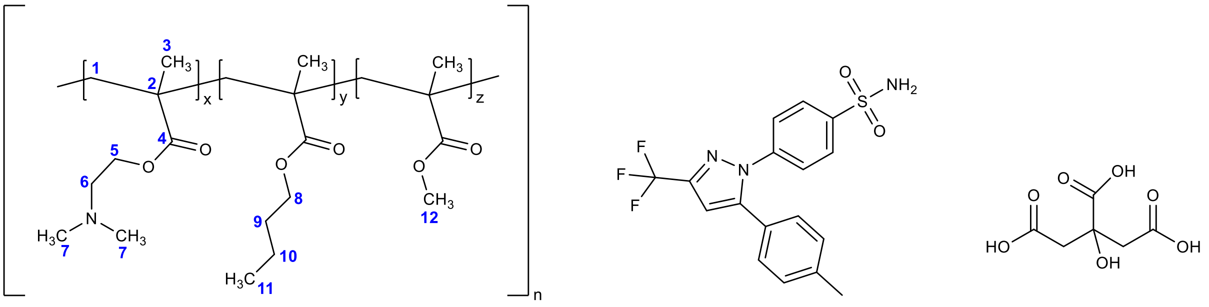

Eudragit® E100 and PO **celecoxib** **citric acid**

Figure S4: Chemical structures of EPO, AA, and CXB, as well as the EPO structure annotated with the corresponding proton positions for the 1H-NMR spectrum.

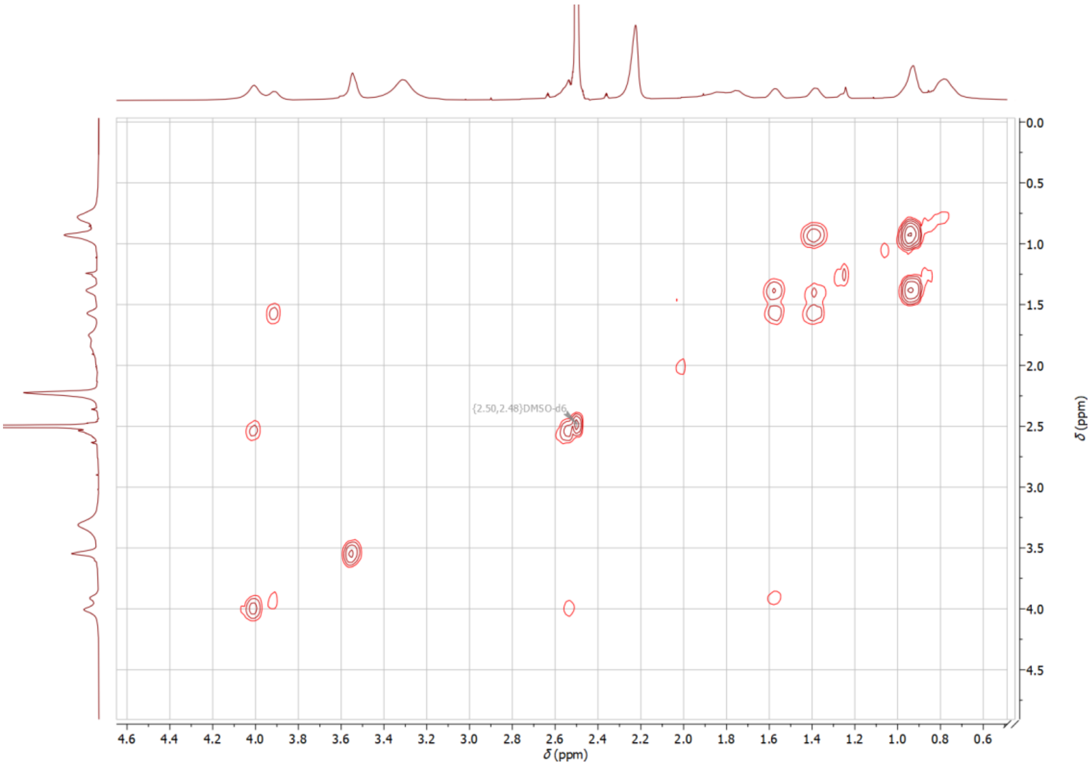

Figure S5: ^{1}H-^{1}H COSY spectra (500 MHz, DMSO-d$_6$, 298K) of EPO.

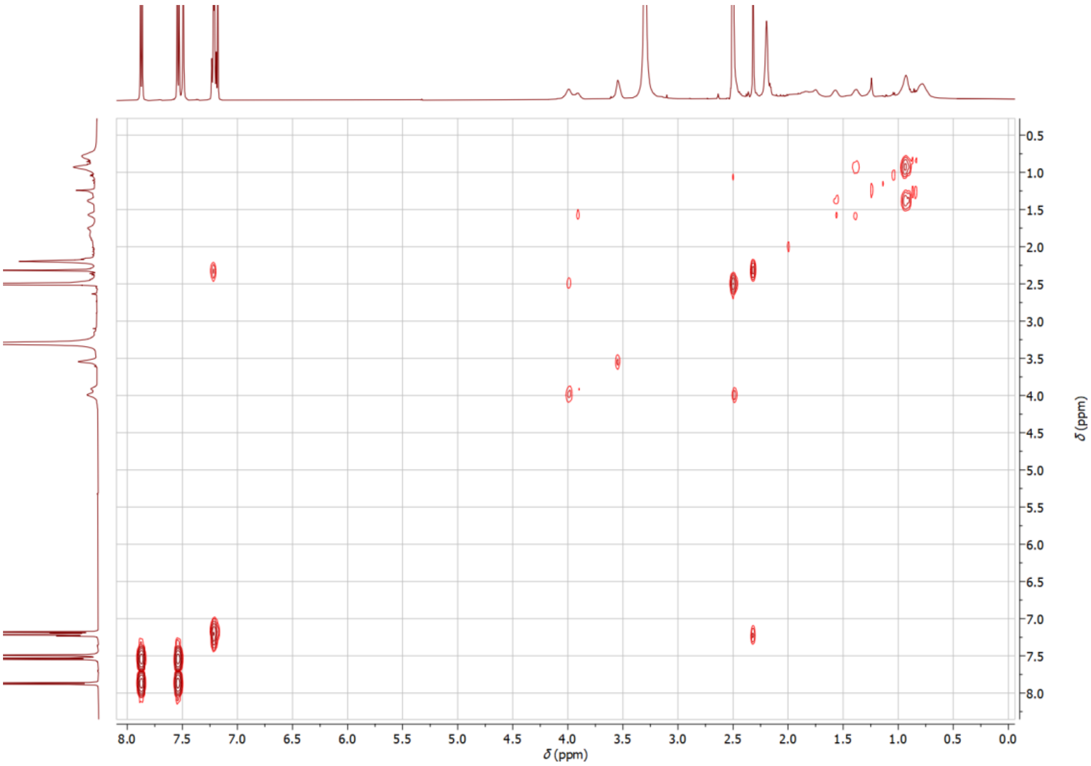

Figure S6: ^{1}H-^{1}H COSY spectra (500 MHz, DMSO-d_6, 298K) of EPO with CXB.

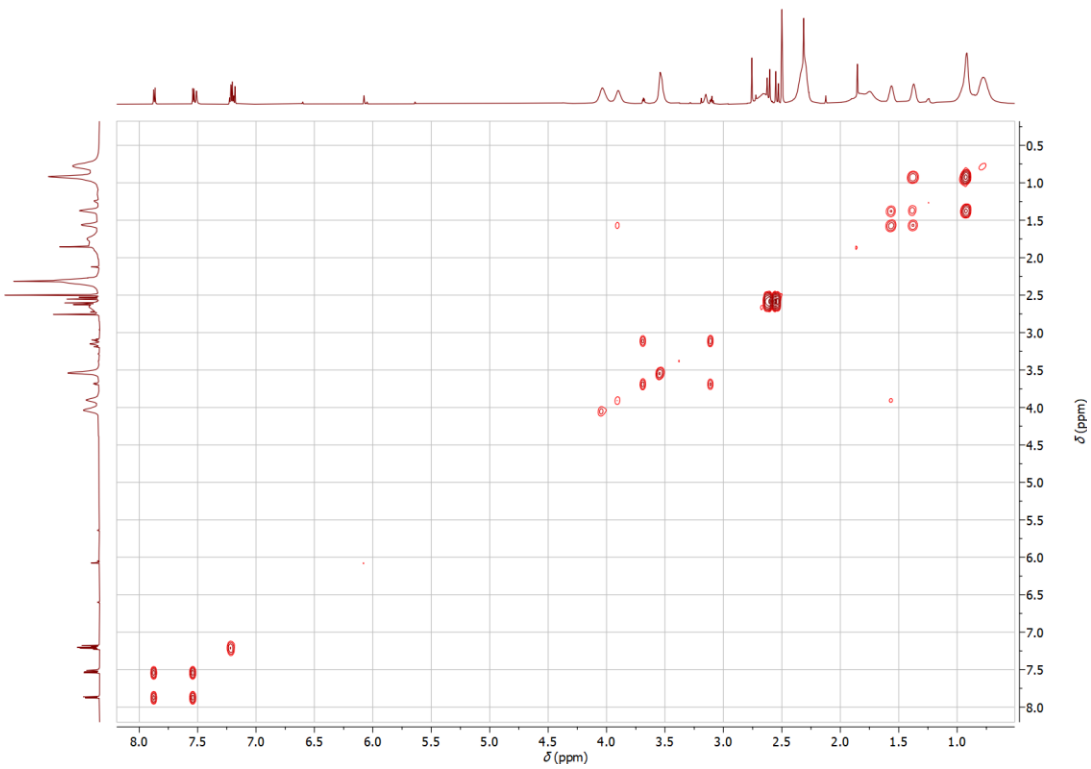

Figure S7: ^{1}H-^{1}H COSY spectra (700 MHz, DMSO-d_6, 298K) of EPO with CXB and CA.

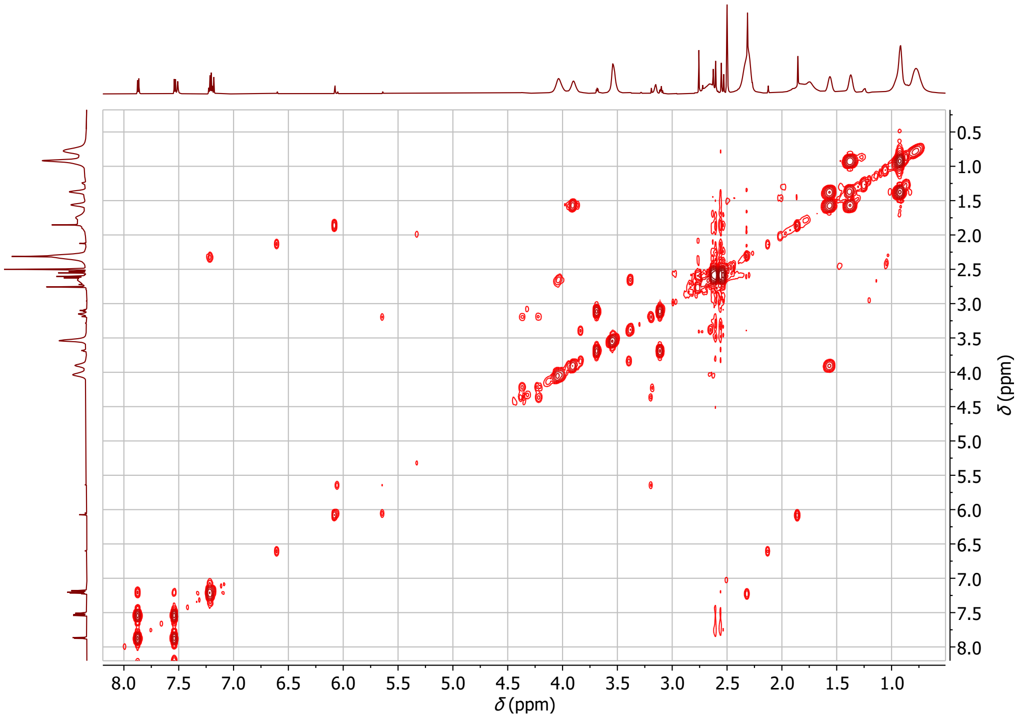

Figure S8: ^{1}H-^{1}H COSY spectra (700 MHz, DMSO-d_6, 298K) of EPO with CXB and CA.

A 8 Non-Sink Dissolution studies

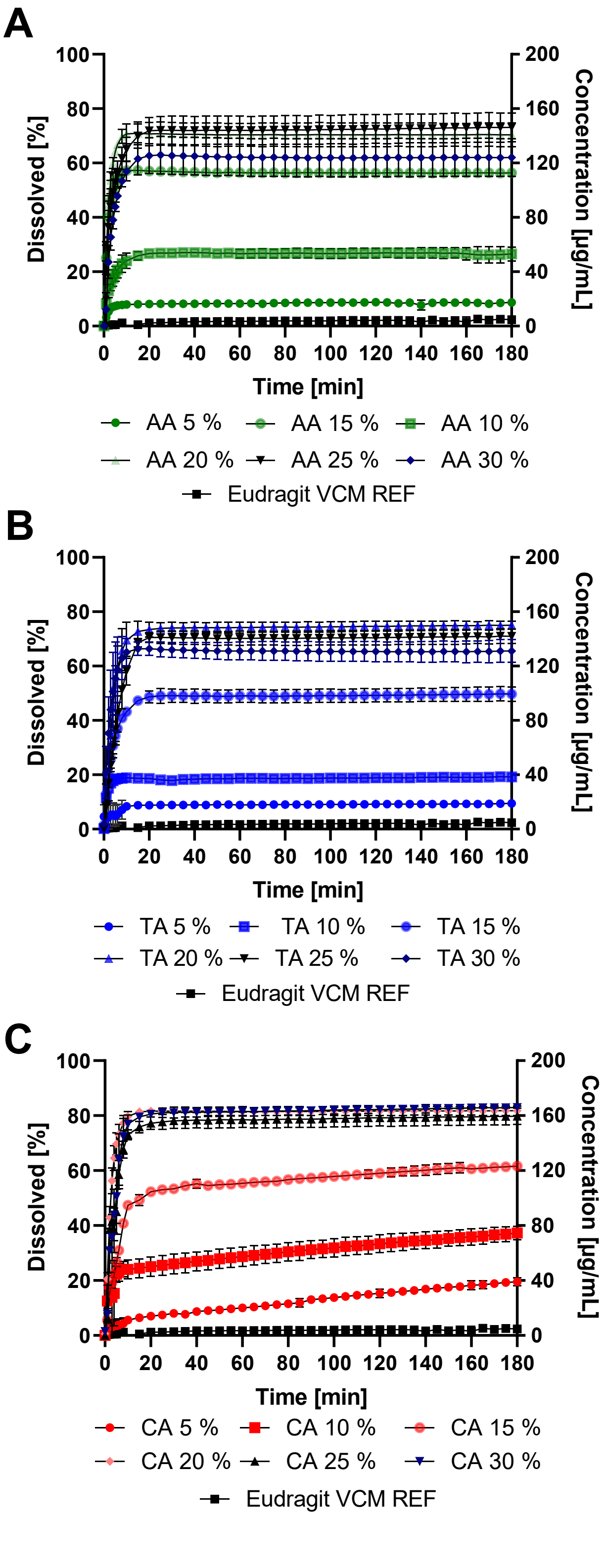

Figure S9: Dissolution profiles of EPO-CXB ASDs with 10% DL and variation in acid content (neat, 5%, 10%, 15%, 20%, 25%, 30%) (A) with AA, (B) AA and (C) TA. Non-sink dissolution study was conducted in 20 ml 0.05 M phosphate buffer at pH 6.8 (37 ◦C, 75 rpm paddle speed). Data are presented as mean ± standard deviation (SD) of three independent measurements (n = 3).

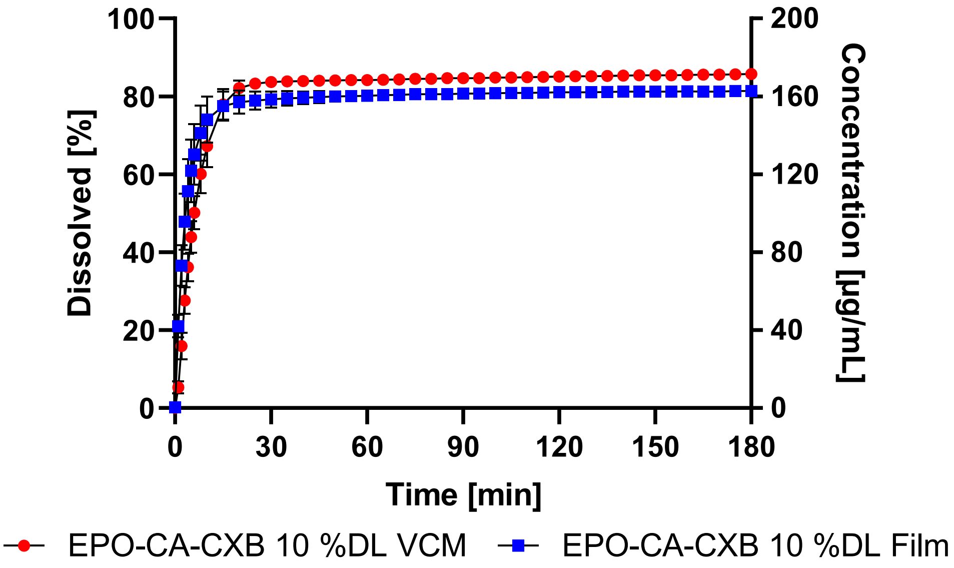

Figure S10: Dissolution profiles of EPO-CA CXB ASDs with 10% DL. Non-sink dissolution study was conducted in 20 ml 0.05 M phosphate buffer at pH 6.8 (37 ◦C, 75 rpm paddle speed). Data are presented as mean ± standard deviation (SD) of three independent measurements (n = 3).

A 9 Particle Size Analysis during Dissolution

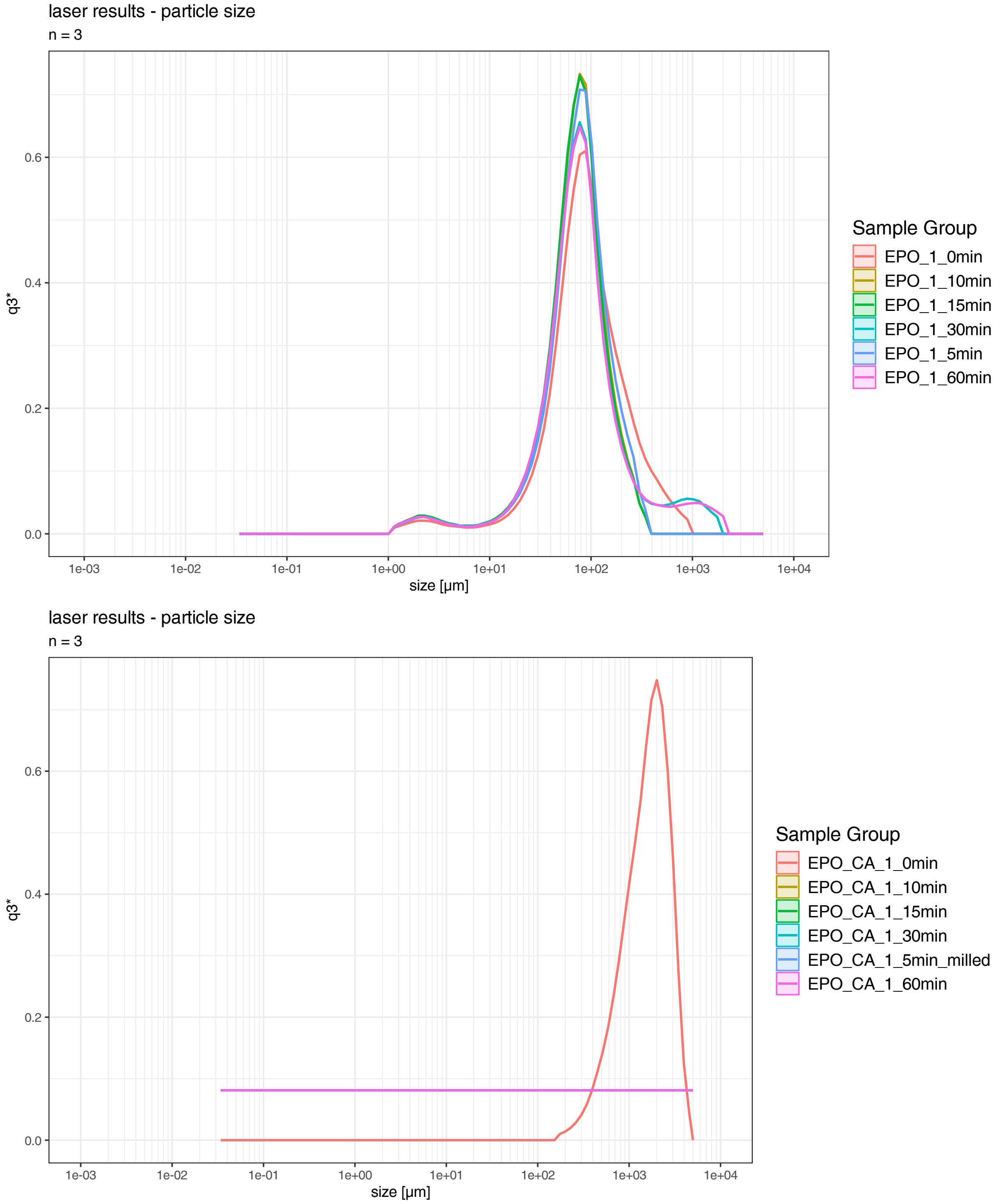

Figure S11: Volume-based particle size distributions (q^{3*}) of EPO and EPO–CA particles measured by laser diffraction during dispersion in PBS at different time points (0–60 min). Values represent mean (n = 3).

A 10 Stability

Physical stability

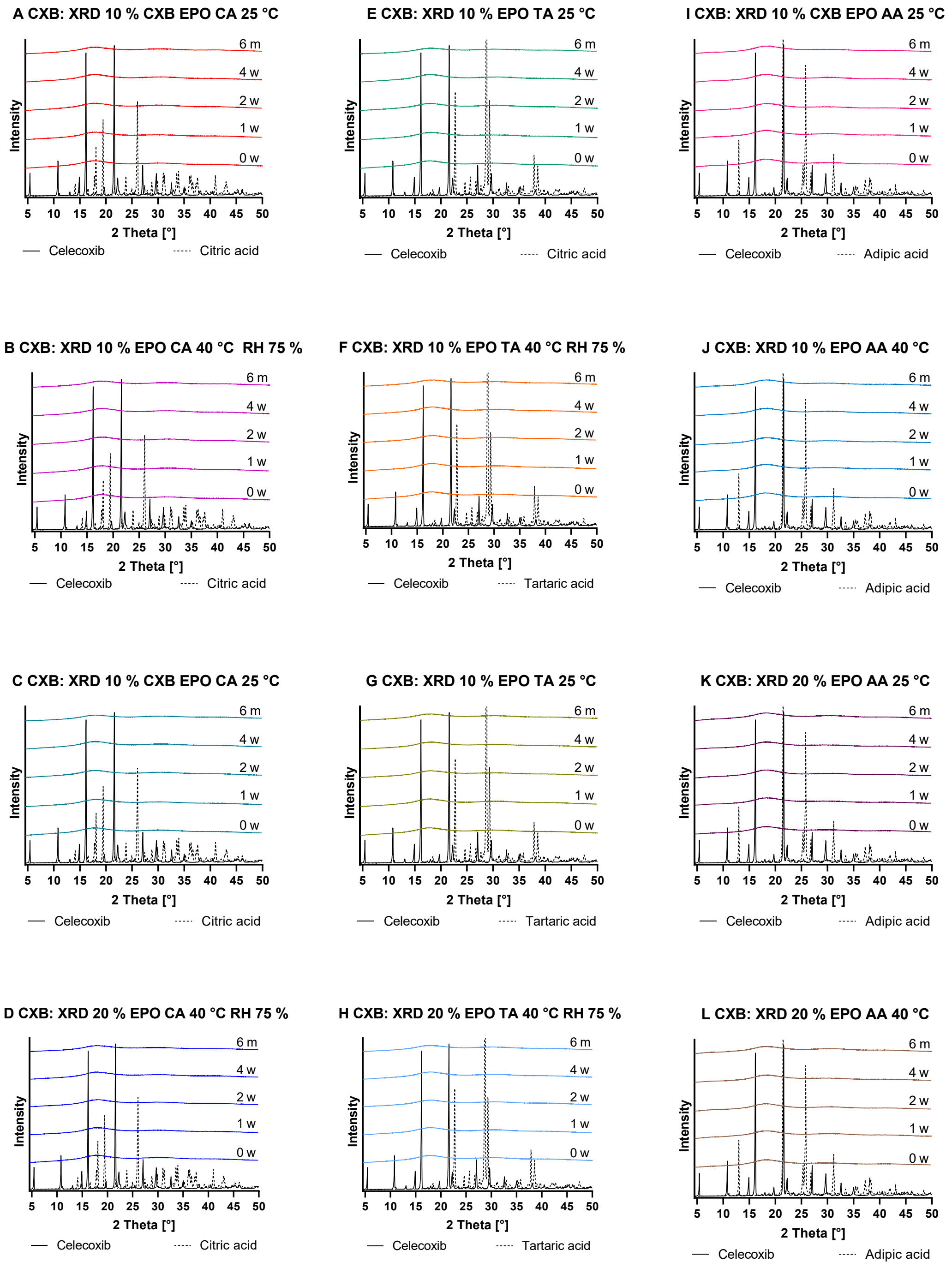

Figure S12: XRPD patterns of EPO-based ASDs (polymer:coformer 80:20) containing 10 % and 20 % DL-CXB with citric acid (CA; A–D), tartaric acid (TA; panels E–H), and adipic acid (AA; I–L), stored in closed glass vials with desiccant according to ICH guidelines. Samples were stored for up to six months at 25 °C / 60 % RH (A, B, E, F, I, J) and under accelerated conditions at 40 °C / 75 % RH (C, D, G, H, K, L). Solid lines indicate CXB reflections, dashed lines indicate the respective acidic coformer. Diffractograms are shown immediately after manufacture (0 weeks), after 1, 2, and 4 weeks, and after 6 months of storage.

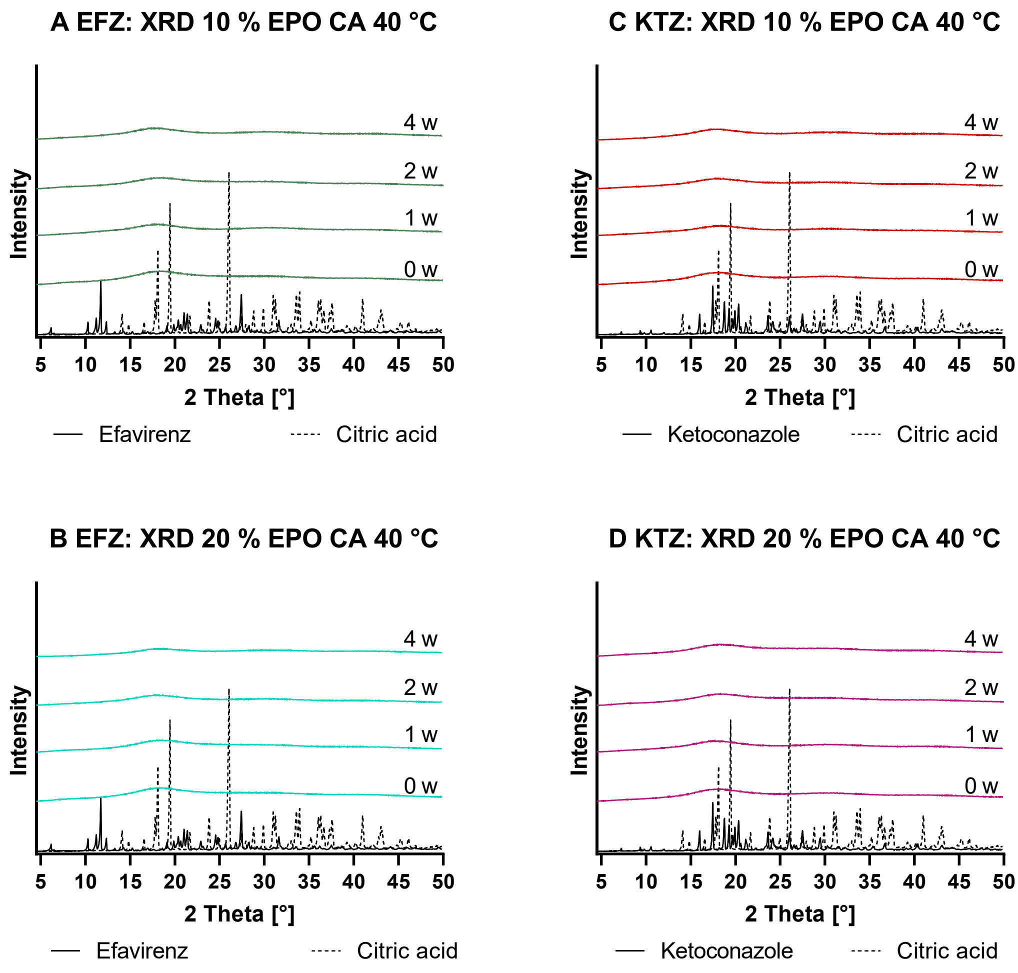

Figure S13: XRPD patterns of EPO-CA-based ASDs (polymer:coformer 80:20) containg 10 % and 20 % DL-EFZ (A, B) and DL-KTZ (C, D) stored under accelerated conditions at 40 °C / 75 % RH in closed glass vials with desiccant according to ICH guidelines. Diffractograms are shown immediately after manufacture (0 weeks) and after 1, 2, and 4 weeks of storage.

Non-Sink Dissolution of stored ASDs

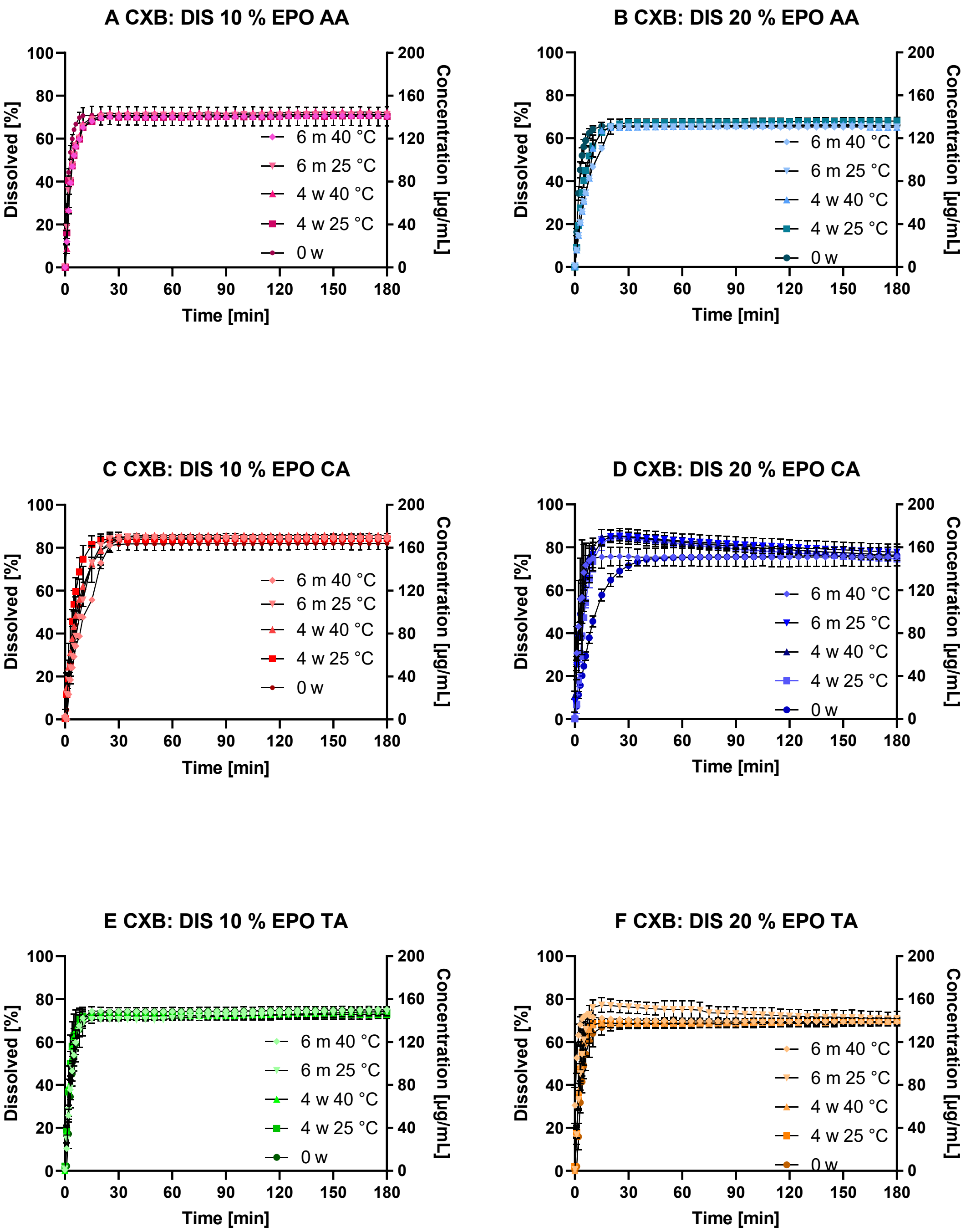

Figure S14: Non-sink dissolution profiles of EPO-based amorphous solid dispersions containing CXB and acidic coformers in 0.05 M phosphate buffer (pH 6.8, 37 °C, 75 rpm) over 180 min. Formulations with 10% DL are shown in (A, C, E) and those with 20% DL in (B, D, F). Each panel depicts ASDs containing a single acid coformer at different storage conditions: AA (A, B), CA (C, D), and TA (E, F). Dissolution profiles are shown for samples measured immediately after manufacture (0 weeks) and after storage under long-term (25 °C / 60% RH) and accelerated (40 °C / 75% RH) conditions in closed glass vials with desiccant, in accordance with WHO guidelines. Data are presented as mean ± SD (n = 3).

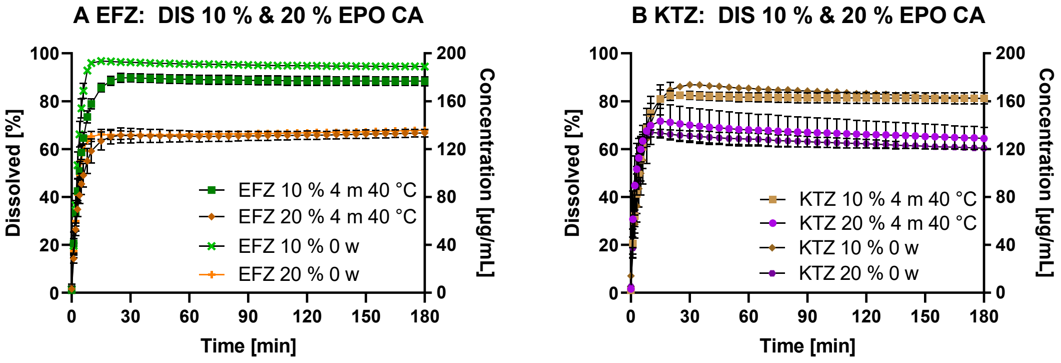

Figure S15: Non-sink dissolution profiles of EPO-CA ASDs containing EFZ (A) and KTZ (B) with acidic coformers in 0.05 M phosphate buffer (pH 6.8, 37 °C, 75 rpm) over 180 min. Samples stored under accelerated conditions (40 °C / 75% RH) in closed glass vials with desiccant in accordance with WHO guidelines are shown. Dissolution profiles are presented for samples measured immediately after manufacture (0 weeks) and after 4 months of storage. Data are shown as mean ± SD (n = 3).
